# Contrastive signal–dependent plasticity: Self-supervised learning in spiking neural circuits

**DOI:** 10.1126/sciadv.adn6076

**Published:** 2024-10-23

**Authors:** Alexander G. Ororbia

**Affiliations:** Department of Computer Science, Rochester Institute of Technology, 1 Lomb Memorial Dr, Rochester, NY 14623, USA.

## Abstract

Brain-inspired machine intelligence research seeks to develop computational models that emulate the information processing and adaptability that distinguishes biological systems of neurons. This has led to the development of spiking neural networks, a class of models that promisingly addresses the biological implausibility and the lack of energy efficiency inherent to modern-day deep neural networks. In this work, we address the challenge of designing neurobiologically motivated schemes for adjusting the synapses of spiking networks and propose contrastive signal–dependent plasticity, a process which generalizes ideas behind self-supervised learning to facilitate local adaptation in architectures of event-based neuronal layers that operate in parallel. Our experimental simulations demonstrate a consistent advantage over other biologically plausible approaches when training recurrent spiking networks, crucially side-stepping the need for extra structure such as feedback synapses.

## Teaser

A neurobiologically plausible recurrent spiking neural network and a dynamic process for adjusting its synapses are proposed.

## INTRODUCTION

The notion of “mortal computation” (*1*–*3*) challenges one of the foundational principles upon which general purpose computers have been built. Specifically, computation, as it is conducted today, is driven by the strong separation of software from hardware. This means that the knowledge contained within a program written in the software is “immortal,” allowing it to be copied to different physical copies of the hardware itself. Machine learning models, which can be viewed as programs that adjust themselves in accordance with data, also rely on this separation. Mortal computation, in contrast, means that once the hardware medium fails or “dies,” the information encoded within it will also disappear, much akin to what would happen to the knowledge acquired by a biological organism when it is no longer able to maintain homeostasis. Despite the transience that comes with the binding of software to hardware, a valuable property emerges—energy efficiency. This motivates a move away from Red AI, the result of immortal computation, and toward Green AI (*4*), addressing a key concern: How might we design intelligent systems that do not escalate computational and carbon costs (*4*–*6*)?

A promising pathway to mortal computation centers around a family of brain-inspired computational models known as spiking neural networks (SNNs) (*7*–*10*)—neuronal “software” that processes and transmits information via discrete electrical pulses—in tandem with hardware such as memristor systems (*11*, *12*) and neuromorphic chips (*13*, *14*). In particular, it has been shown that neuromorphic-instantiated SNNs can be several orders of magnitude more power efficient (*15*, *16*) than modern-day deep neural networks (*17*). This type of in silico mortal computation could bring to machine intelligence elements of the human brain’s comparatively minimal energy consumption (*18*). However, a challenge facing the development of these types of systems is the design of the requisite architectures and “credit assignment” schemes, i.e., algorithms that determine the positive/negative contributions of each neuron to an SNN’s overall behavioral improvement, that could be cast in terms of in-memory processing (*19*, *20*)—where memories correspond to the SNN’s synaptic connections—to side-step the “von Neumann bottleneck” (*19*) or the extra thermodynamic costs induced by reading and writing to memory.

In service of the efficiency afforded by a neuromorphic mortal computer, some of the key qualities that should characterize its underlying inference and learning computations include: (i) no requirement for differentiability in the spike-based communication [backprop through time as applied to SNNs requires differentiability and thus the careful design of surrogate functions (*21*, *22*)], (ii) no forward-locking (*23*) in the propagation of information through the system (a layer of neurons can compute their activities without waiting on other layers to update their own), (iii) no backward-locking (*23*) in the synaptic updating phase (changes to synapses for one layer of neurons can be executed in parallel with other layers across time—updates are local in both space and time), and (iv) no requirement for feedback synaptic pathways (*24*), especially those that traverse backward along the same forward propagation pathways (weight transport) (*25*), to calculate synaptic change [frameworks such as spiking predictive coding require feedback pathways to carry out the requisite message passing (*26*–*28*)].

In this study, we satisfy the above criteria through the development of a biologically plausible neural circuit and a self-supervised learning scheme that formulates forward-only (*29*) and forward-forward (FF) credit assignment (*1*, *2*) for spike-based neuronal communication. FF learning, in general, formulates the adaptation of the parameters of an artificial neural network (ANN) as a process of manipulating the probabilities that it assigns to the data points presented to it, i.e., the goal is for the ANN to raise the probability for actual data while lowering the probability for fake data. Adjustments are then made, under FF, to the synaptic weights of the ANN based on these probabilities in the context of a scoring objective assigned to each of its layers. The principles underlying FF adaptation motivate this work’s central contributions, which include the following

1) The design of a recurrent spiking neural circuit that exhibits layer-wise parallelism, where each layer is driven by top-down, bottom-up, and lateral pressures that do not require feedback synaptic pathways across the network—this means that our learning and inference processes directly resolve the forward and update locking problems;

2) The proposal of contrastive signal–dependent plasticity (CSDP) for adapting the synapses of spiking neural systems in a dynamic fashion suitable for in-memory computation, further offering a complementary rule to spike timing–dependent plasticity (STDP) (*30*);

3) The development of simple, fast neural circuit mechanisms that locally learn to classify and/or reconstruct activity signals; and

4) A quantitative evaluation of the generalization ability of our CSDP-learned SNNs.

## RESULTS

### A recurrent spiking neural circuit

**Fig. 1.**
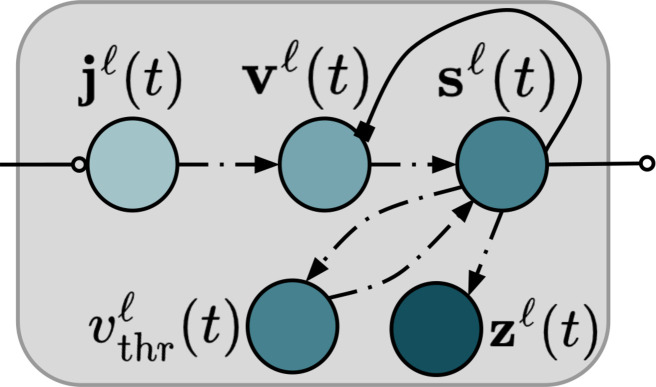
Componential layout of one layer in the CSDP SNN recurrent circuit. A component diagram of a leaky integrate-and-fire (LIF) cell group/vector. The neuronal components include the electrical current **j**^*ℓ*^(*t*), the membrane voltage potential **v**^*ℓ*^(*t*), the spike emission **s**^*ℓ*^(*t*), the activation trace **z**^*ℓ*^(*t*), and the cross-layer homeostatically constrained threshold $v_{\mathrm{thr}}^{\ell }\left(t\right)$. Black (dashed) arrows with solid triangle heads indicate the flow of values within an LIF group’s dynamics [e.g.., current **j**^*ℓ*^ is input to dynamics of **v**^*ℓ*^(*t*)], open-circle heads indicate excitation/additive pressure, and solid square heads indicate inhibitory/subtractive pressure.

The neuro-mimetic architecture designed in this work is made up of recurrent layers that operate in parallel with each other (*L* layers in total), where each layer *ℓ* contains *J*_*ℓ*_ neuronal cells, which are further made up of multiple components (see Fig. 1). A natural by-product of our neural circuit design is that all layers of spiking neuronal cells can be simulated, at each step in time, completely in parallel, further permitting possible implementations with neural layers that are executed asynchronously, e.g., such as in coupled neuromorphic crossbars. Specifically, at time *t*, each layer *ℓ* of neurons takes in as input the previous (spike) signals emitted (at time *t* − 1) from the neurons immediately below (from layer *ℓ* − 1), i.e., $s^{\ell –1}(t)\in {{\left\{0,1\right\}}^{J_{\ell –1}}}^{\times 1}$, as well as those immediately above (from layer *ℓ* + 1), i.e., $s^{\ell +1}(t)\in {{\left\{0,1\right\}}^{J_{\ell +1}}}^{\times 1}$. **W**^*ℓ*^ is the bundle of synaptic connections that propagate information from the layer below (*ℓ* − 1), and **V**^*ℓ*^ is the synaptic bundle that transmits information from the layer above (*ℓ* + 1). Furthermore, the neurons in each layer are laterally connected to one another, where the synapses **M**^*ℓ*^ that connect them enforce a form of dynamic inhibition: Neurons that are more strongly active will suppress the activities of the more weakly active ones in **s**^*ℓ*^(*t*). This results in an emergent form of *K* winners-take-all competition, where *K* changes with time. Last, we explore the integration of a set of optional top-down “attentional” class-mediating (spike) signals **s***_y_* ∈ {0, 1}^*C*×1^ (where *C* is the number of classes), transmitted along the synaptic bundle **B**^*ℓ*^. These encourage spiking neural units to form representations that encode any available discriminative information, e.g., labels/annotations. Incorporating these class contextual synapses results in a supervised model.

**Fig. 2.**
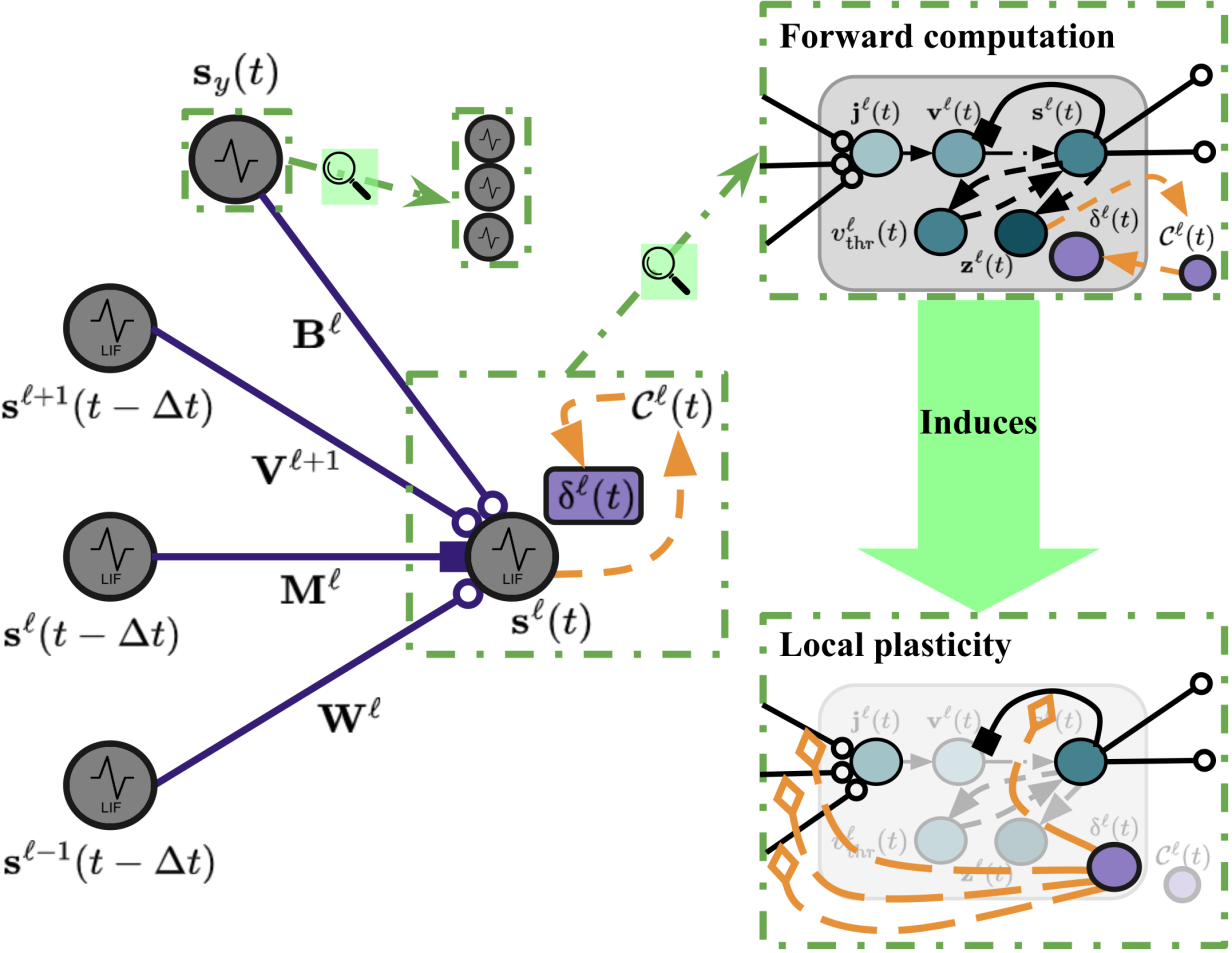
Neuronal layer within a CSDP-trained recurrent SNN. Depicted is the neural computation underlying one layer of our recurrent spiking circuit as well as its synaptic adjustment, induced via CSDP. Note that the supervised variant of CSDP uses class modulation synapses **B**^*ℓ*^, whereas the unsupervised variant does not. To facilitate contrastive learning, we integrate another biochemical component δ^*ℓ*^(*t*) into our leaky integrator cell model. Once electrical current **j**^*ℓ*^(*t*) has been injected into the cells, triggering an update to their membrane potentials **v**^*ℓ*^(*t*), possibly leading to emission of action potentials **s**^*ℓ*^(*t*), their glutamate traces contribute to a goodness modulator $C^{\ell }\left(t\right)$ which then deposits a local synaptic adjustment signal to each cell’s $\delta_{i}^{\ell }\left(t\right)$ component. Solid lines indicate synaptic pathways; those with open-circle heads indicate excitation/additive pressure, solid square heads indicate inhibitory/subtractive pressure, and diamond heads indicate modulation/multiplicative pressure. Dashed lines (with solid triangle heads) indicate nonsynaptic pathways (where no transformation is applied).

Concretely, the dynamics of any single layer of neuronal processing units (NPUs) or cells within our biomimetic model follows that of the leaky integrator (*31*), and the full dynamics (see Fig. 2 for a visual depiction) of any layer of spiking NPUs is calculated in terms of electrical current **j**^*ℓ*^(*t*) as follows

$$\begin{array}{c} d_{i}^{\ell }\left(t\right)=R_{E}\left[\Sigma_{j=1}^{J_{y}}W_{ij}^{\ell }s_{j}^{\ell -1}\left(t\right)\right]+R_{E}\left[\Sigma_{j=1}^{J_{\ell +1}}V_{ij}^{\ell }s_{j}^{\ell +1}\left(t\right)\right]\\ -R_{I}\left\{\Sigma_{j=1}^{J_{\ell }}\left[M_{ij}^{\ell }\left(1-I_{ij}^{\ell }\right)\right]\cdot s_{j}^{\ell }\left(t\right)\right\} \end{array}$$ (1)

$$j_{i}^{\ell }\left(t\right)=\{\begin{array}{cc} d_{i}^{\ell }\left(t\right) & y=\emptyset \\ d_{i}^{\ell }\left(t\right)+R_{E}\left[\Sigma_{j=1}^{J_{y}}B_{ij}^{\ell }\cdot s_{y,j}\left(t\right)\right] & y\neq \emptyset \end{array}$$ (2)

which then triggers an update to the NPUs’ membrane voltage values **v**^*ℓ*^(*t*)

$${\hat{v\;}}_{i}^{\ell }\left(t+\Delta t\right)=v_{i}^{\ell }\left(t\right)+\left(\Delta t/\tau_{m}\right)\left[-v_{i}^{\ell }\left(t\right)+j_{i}^{\ell }\left(t\right)\right]$$ (3)

and, lastly, results in the emission of spikes **s**^*ℓ*^(*t*) (or action potentials)

$$\begin{array}{c} s_{i}^{\ell }\left(t+\Delta t\right)={\hat{v}}_{i}^{\ell }\left(t+\Delta t\right)>v_{\text{thr}}^{\ell },\;\text{and},\\ v_{i}^{\ell }\left(t+\Delta t\right)={\hat{v}}_{i}^{\ell }\left(t+\Delta t\right)\left[1-s_{i}^{\ell }\left(t+\Delta t\right)\right] \end{array}$$ (4)

$$v_{\text{thr}}^{\ell }=v_{\text{thr}}^{\ell }+\lambda_{v}\left\{\left[\Sigma_{j=1}^{J_{\ell }}s_{j}^{\ell }\left(t+\Delta t\right)\right]-1\right\}$$ (5)

where (1 − **I**^*ℓ*^) creates a *J*_*ℓ*_ × *J*_*ℓ*_ hollow matrix. The hollow matrix is used to ensure that there is only cross-inhibition in any layer *ℓ* and no self-excitation/inhibition [if one wanted to explicitly model self-inhibitory/excitatory effects, one could either remove the (1 − **I**^*ℓ*^) to place self-inhibition or add an extra term (+α**I**) to incorporate self-excitation; we leave this for future work]. Equation 2 states that, for any layer *ℓ* at time *t*, the electrical current input **j**^*ℓ*^(*t*) is a function of spatially near (i.e., bottom-up, top-down, and lateral) as well as (possibly existent) target class-mediating action potentials. In Eq. 3, **v**^*ℓ*^(*t*), the vector containing the membrane voltage potential values for each cell, is driven by the current produced by Eq. 2 [where excitatory currents are weighted by resistance constant *R*_E_ in (deci)ohms and inhibitory currents are weighted by inhibitory resistance *R*_I_]. This voltage ultimately triggers the emission of a layer’s output spike **s**^*ℓ*^(*t*), as in Eq. 4. Δ*t* is the integration time constant on the order of milliseconds, while τ*_m_* is the membrane time constant (in milliseconds). Further notice that our spike emission model (Eq. 4) entails a depolarization of the membrane potential: This (re)sets the voltage to a resting potential of 0 (deci)volts through binary gating. Note that, while we omit modeling refractoriness for simplicity, our dynamics are flexible enough to warrant incorporating both relative and absolute refractory periods to promote additional sparsity.

In Eq. 5, we simulate an adaptive, nonnegative spiking threshold ${v_{\text{thr}}}^{\ell }\in \left[0,\infty \right)$ which, at time *t*, either results in the addition or subtraction of a small value—scaled by λ*_v_*, which is a constant typically set to a number such as 0.001—from a current threshold value based on how many spikes were recorded at time *t* for layer *ℓ*. This represents a simple homeostatic constraint on short-term plasticity, encouraging each layer to emit as few spikes as possible at any step in time. Last, notice that **s**^0^(*t*) is the binary spike representation of the sensory input **x** at time *t*. A spike is created from this pattern vector, e.g., an image, by sampling the normalized pixel values of **x** (each value/element of **x** is divided by a scalar, such as the maximum pixel value is 255), treating each value as Bernoulli probability. In this study, **s***_y_*(*t*) is the target class spike train associated with **x**. If a label is available, then **s***_y_*(*t*) = **y** (at each time step, it is clamped to the sparse label during training), otherwise **s***_y_*(*t*) = ∅ (since **y** = ∅).

Notice that the key property of the recurrent spiking system above is that, by design, it is layer-wise parallelizable and therefore naturally not forward-locked (*23*). In essence, each layer’s spike emissions can be computed in parallel to the other layers. In addition, as will be seen later, the system is not update/backward-locked—changes in synaptic strengths for any layer can be computed in parallel of the others—due to the fact that our plasticity scheme operates with information that is local in both time and space. This stands in contrast to many (deep) SNN designs today, where each layer of spiking NPUs depends on the previous spike activities of the layer that comes before them. Other models that are not forward-locked, such as spiking predictive coding schemes (*26*–*28*), generally rely on introducing additional neuronal circuitry in the form of feedback cycles which increases the complexity of the neural computation that underlies inference. Our model ensures an architectural parallelism by only enforcing the leaky integrator spike activities to depend on recently computed spatially local activities, which is biologically more plausible as well as efficient and cheap to compute.

### Plasticity dynamics: CSDP

Key to the long-term plasticity process that we propose in this study is the trace variable. Practically, this will mean that an additional component, responsible for maintaining what is called an “activation trace,” is introduced to each neuronal cell of the recurrent spiking system. As a result, for each layer, a trace is dynamically updated in the following manner

$$z_{i}^{\ell }\left(t+\Delta t\right)=z_{i}^{\ell }\left(t\right)+\left(\Delta t/\tau_{\text{tr}}\right)\left[-z_{i}^{\ell }\left(t\right)+\gamma s_{i}^{\ell }\left(t+\Delta t\right)\right]$$ (6)

where, in this work, the trace time constant was set to τ_tr_ = 3 ms and γ = 0.05. An activation trace smooths out and filters the sparse spike train signals generated by each layer of NPUs while still being biologically plausible. Mechanistically, a trace offers a dynamic rate-coded equivalent value that, in our model, would likely be biophysically instantiated in neuronal cells in the form of concentrations of internal calcium ions (*32*). Desirably, this will allow our adaptation rule to take place using (filtered) spike information, bringing it closer to a form of STDP-like adjustment (*30*) rather than directly operating on voltage or electrical current values, as in related efforts in developing credit assignment processes for SNNs (*33*, *34*).

**Fig. 3.**
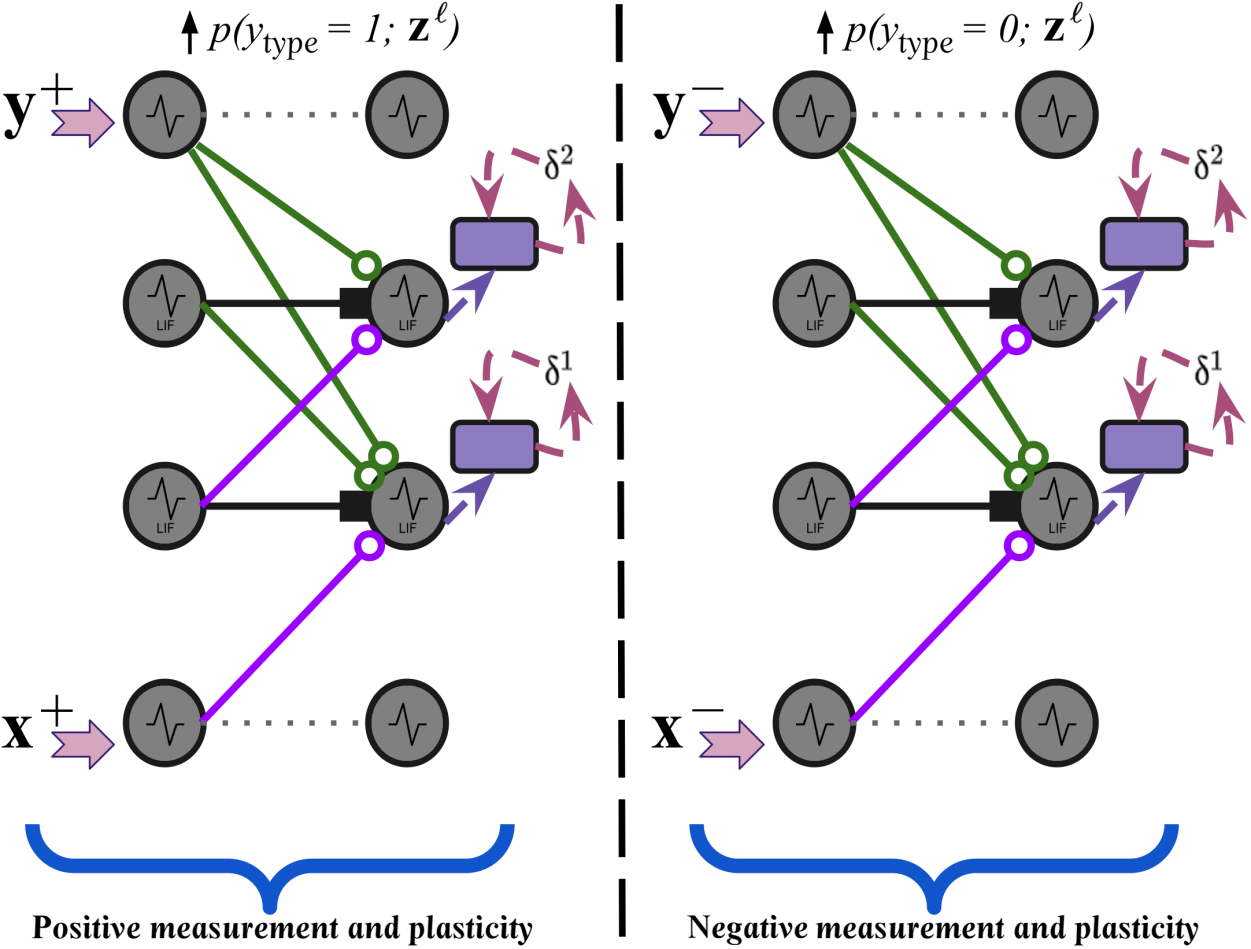
Positive and negative modes of CSDP plasticity in a recurrent SNN. Shown are the two parallel modes of plasticity that our recurrent spiking circuit undergoes—a positive measurement mode is induced when a sensory input and its corresponding (if available) target class are used to drive inference and plasticity (this entails neural layers increasing the probability values assigned to a sensory pattern), and a negative measurement mode is induced when a negative/out-of-distribution sample and its corresponding negative target class are used to drive the same underlying neural calculations (this entails neural layers lowering probabilities that they ultimately assign). Solid square arrows denote inhibitory pressure, whereas open-circle arrows denote excitatory pressure. Colors for different synaptic pathways denoted by solid lines, all of which are plastic, are provided to visually distinguish between top-down class modulating synapses (green), bottom-up sensory driving synapses (purple), and lateral synapses (black).

Given the aforementioned activation trace, the objective that each layer of our spiking system will try to optimize, at each step of simulated time, may next be specified. Specifically, in our plasticity process, CSDP, we propose adjusting SNN synaptic strengths by integrating the “goodness principle” inherent to FF learning (*1*, *2*) into the dynamics of spiking neurons. This, in turn, will mean that any layer of NPUs will work to raise the probability they (collectively) assign to the incoming presynaptic messages that they receive if the sensory input comes from the environment (it is “positive”). Conversely, NPUs will strive to lower the probability that they assign if the input is fake/nonsensical (it is “negative” or out of distribution); see Fig. 3. As a result, the (local) contrastive function takes the following form

$$\begin{array}{c} C\left[z^{\ell }\left(t\right),y_{\text{type}}\right]=\\ -\left\{\overset{\text{Positive Term}}{\overbrace{y_{\text{type}}\text{log}p\left[y_{\text{type}}=1;z^{\ell }\left(t\right)\right]}}+\overset{\text{Negative Term}}{\overbrace{\left(1-y_{\text{type}}^{\ell }\right)\text{log}p\left[y_{\text{type}}=0;z^{\ell }\left(t\right)\right]}}\right\} \end{array}$$ (7)

where the goodness probability *p*(*y*_type_ = 1; **z**^*ℓ*^) that will be computed by a group of spiking neurons in any layer *ℓ* can be directly defined as

$$p\left[y_{\text{type}}=1;z^{\ell }\left(t\right)\right]=1/\left[1+\text{exp}\left(-\left\{\Sigma_{k=1}^{J_{\ell }}{\left[z_{k}^{\ell }\left(t\right)\right]}^{2}-\theta_{z}\right\}\right)\right]$$ (8)

Note that *y*_type_ ∈ {0, 1} is an integer which denotes what “type” of sensory input a sample of incoming (presynaptic) activity signals feeding into (postsynaptic) layer *ℓ* originate from, i.e., 1 indicates that such signals originate from or are caused by positive (in-distribution) sensory input, while 0 indicates that they come from negative (out-of-distribution) input. θ*_z_* is a fixed threshold against which the square of the trace values are compared and is set to the value θ*_z_* = 10. As a result of the local contrastive function (Eq. 7), the adjustments made to synaptic bundles for any layer become simple, efficient modulated Hebbian-like rules (see Supplementary Text for derivations and connections made to STDP). For instance, with respect to the synapses **W**^*ℓ*^ connecting the layer below *ℓ* − 1 to layer *ℓ*, the resultant update would be

$$\delta_{i}^{\ell }\left(t\right)=\partial C\left[z^{\ell }\left(t\right),y_{\text{type}}\right]/\partial z_{i}^{\ell }\left(t\right)$$ (9)

$$\Delta W_{\mathrm{ij}}^{\ell }=\left[R_{m}\delta_{i}^{\ell }\left(t\right)s_{j}^{\ell -1}\left(t-\Delta t\right)\right]+\lambda_{d}\left\{s_{i}^{\ell }\left(t\right)\left[1-s_{j}^{\ell -1}\left(t-\Delta t\right)\right]\right\}$$ (10)

where λ_d_ is the synaptic decay factor and δ^*ℓ*^(*t*) ∈ $R^{J_{\ell }\times 1}$ (a vector containing one modulatory signal per neuron). See Materials and Methods for the updates with respect to all possible sets of synapses. Ultimately, the updates to all four possible synaptic bundles that transmit information to and drive the dynamics of layer *ℓ* depend on the easily computed partial derivative(s) δ^*ℓ*^(*t*) of the local contrastive function $C\left[z^{\ell }\left(t\right),y_{\text{type}}\right]$. We postulate that the vector signal δ^*ℓ*^(*t*), scaled by the resistance, would be biologically implemented either in the form of a (neurotransmitter) chemical signal or, alternatively, as a separate population of NPUs that synthesize this signal, much akin to the error neurons that characterize predictive processing (*35*). Furthermore, the above synaptic plasticity equations could be viewed as a special kind of multifactor Hebbian rule where the modulator term is deposited from another locally embedded neural component (within any one NPU) that biochemically signals the contrastive function $C\left[z^{\ell }\left(t\right),y_{\text{type}}\right]$, as opposed to the typical temporal (reward) difference error found in three-factor Hebbian/STDP update schemes. See Fig. 2 for a depiction of the mechanics that underpin CSDP. Our local contrastive modulatory factor desirably stands on the more recent empirical evidence found in support of self-supervised learning in neurobiological systems (*36*, *37*). The two general “modes” of plasticity that our system undergoes are depicted in Fig. 3. Specifically, if an in-distribution sensory pattern (and possibly any corresponding class target vector) is fed in, then the neuronal system tries to increase the probability assigned to the pattern, whereas for an out-of-distribution pattern, it tries to decrease this probability.

Holistically, our neural dynamics and plasticity process could be treated as operating under a total goodness contrastive functional (function of functions) over the entire neural circuit. Specifically, this objective entails viewing our neural circuit as optimizing, iteratively across a span of time, the following

$$E\left(\theta \right)=\sum_{t=1}^{T}F\left(t,\Theta \right)=\sum_{t=1}^{T}\sum_{\ell =1}^{L}C\left[z^{\ell }\left(t\right),y_{\text{type}}\right]$$ (11)

where Θ is a construct that stores every single bundle of synapses (see Materials and Methods), and $C\left[z^{\ell }\left(t\right),y_{\text{type}}\right]$ is the local goodness function as defined in Eq. 7 (from which synaptic updates for synapses related to layer *ℓ* come from; see Supplementary Text for details). Total goodness is effectively an aggregation of all of the goodness measurements produced by every layer of the neural circuit. In effect, a full recurrent spiking circuit, under our framework, is optimizing, at time *t*, its distributed representations of sensory inputs with respect to a sequence loss E(Θ) which sums, over all timesteps, the total system-level goodness values F(*t*, Θ).

### Synthesizing negative patterns for supervised and unsupervised CSDP

Given that CSDP inherently centers around self-supervised contrastive adaptation, a process for producing negative or out-of-distribution data patterns to contrast with encountered sensory patterns must be provided. Note that, on the basis of the dynamics presented above, two general variants of CSDP are possible: a supervised variant if the class-modulating synapses **B**^*ℓ*^ are used and an unsupervised variant if they are not. Depending on which variant is used, a different scheme is used for generating out-of-distribution/negative data points that further operated on-the-fly, i.e., only used information/statistics within a current batch or small buffer of pooled data samples.

In the case of the supervised model, we adapted the scheme used in (*1*, *2*). For each (**y**, **x**) in a minibatch/pool of patterns, we would produce a negative batch (**y**^−^, **x**^−^) by duplicating the original images, and for each cloned (original/positive) pattern, we then created a corresponding “incorrect” paired label by randomly sampling a class index other than the correct one (index *c*) found within **y**. Formally, if *c* ∈ {1, …*C*} is the correct class index of **y**, then a negative label, with incorrect class index *q* ≠ *c*, is produced via: **y**^−^ = **1***_q_* where *q* ∼ {1, …, *c* − 1, *c* + 1, …, *C*}. For the unsupervised variant, where **y** = ∅, we took inspiration from (*38*) and designed an on-the-fly process that applied the following steps to a current minibatch of patterns: (i) for each pattern **x**(*i*), we would select one other different pattern **x**(*j*), *j* ≠ *i*, within the batch, (ii) apply a random rotation to **x**(*j*) (by sampling a rotation value, in radians, from the range (π/4, (7π)/4) to create **r**(*j*), and lastly, (iii) produce a negative pattern via the convex combination **x**^−^(*i*) = η**x**(*i*) + (1 − η)**r**(*j*) with η = 0.55 (η interpolates between the original and non-original pattern).

### Task-specific generalization

The full neural circuit described above is effectively a spiking representation learning system. In other words, it does not engage in any particular behavioral task. To extend our circuit so that it exhibits task-specific functionality, we investigated modifications that facilitated sensory pattern reconstruction and label classification. Specifically, to promote reconstruction, we integrated, in each layer *ℓ*, a small bundle of extra synapses that made local predictions of the spike activity of the layer immediately below *ℓ* − 1. These local prediction units also follow the same dynamics as the leaky integrate-and-fire (LIF) layers described earlier, e.g., Eqs. 2 to 4, but instead adapt with an error-driven Hebbian rules. To facilitate classification, we integrated a small spiking subcircuit that directly wired the spike emissions of each (non-input) layer to a single output prediction of the target class spike train **s***_y_*(*t*)—this speeds up the test-time model inference for the fixed memory cost of several extra synaptic matrices. The output units that predicted the class spike train again follow the same LIF dynamics as described before, but their specific synapses, like those of the local reconstruction units, are adapted with error-driven Hebbian rules. See Materials and Methods for the formal details related to our framework’s biophysical generative and classification dynamics and plasticity processes.

### Classification performance

To evaluate the efficacy of our recurrent spiking circuitry and the proposed CSDP scheme, we conducted several experimental simulations in visual symbol recognition, using the Modified National Institute of Standards and Technology (MNIST) and Kuzushiji-MNIST (K-MNIST) datasets. We simulate both unsupervised and supervised variants of the CSDP modeling framework—for unsupervised models, we use the label “CSDP, Unsup,” whereas for supervised models, we use the label “CSDP, Sup”—and compare with several previously reported STDP-adapted SNN results as well as several implemented spiking network biophysical credit assignment baselines. The particular baselines that we implemented and study include the following: (i) a spiking network classifier trained with direct random target propagation (DRTP) (*39*); (ii) a spiking network trained with broadcast feedback alignment (BFA) (*40*); (iii) a spiking network trained with a simplified variant of signal propagation (*29*, *34*) using a voltage-based rule and a local cost based on the Euclidean (L2) distance function (L2-SigProp); (iv) a spiking network trained using a generalization of local classifiers/predictors, where each layer-wise classifier, instead of being held fixed, is learned jointly with the overall neural system (*41*, *42*) (Loc-Pred); and (v) an STDP-trained spiking network (*43*) (STDP-SNN), adapted to operate under this work’s experimental settings [we used the simultaneously trained model architecture of (*43*), which contained one very wide hidden layer of 10,000 LIFs, since Hao *et al.* demonstrated that STDP processes struggle to train networks with more than one hidden layer (*43*), offering no further benefit beyond a single-layer formulation]. Furthermore, we provide a rate-coded baseline model, i.e., one that does not operate with spike trains but instead with rate-coded values, represented by a tuned, backpropagation-trained feedforward neural network (BP-FNN). Notably, SNNs learned via BFA, DRTP, or Loc-Pred require additional neural circuitry to create either feedback loops or layer-wise local predictors, whereas L2-SigProp and the STDP-SNN do not.

The network models simulated under each learning algorithm were designed to have two latent/hidden layers, with each consisting of up to a maximum of 7000 LIF neurons (except for STDP, which has one layer of two laterally wired groups of 10,000 LIFs). We further study and report, in Table 1, for both supervised and unsupervised cases of CSDP, the performance of a “small” CSDP model (labeled as “Sm”), which contained 2250 LIFs in the first layer and 200 LIFs in the second, as well as that of a “large” CSDP model (labeled as “Lg”), which contained 5000 LIFs in the first layer and 1000 LIFs in the second. The smaller model, as a result, is made up of about only 7.8 million synapses (thus, it is comparable to the amount of plastic synapses used in the STDP-SNN baseline), whereas the larger model is made up of about 40 million synapses (thus, it is roughly comparable to the amount of plastic synapses used in the bigger feedback-driven baselines). Synaptic connection efficacies were randomly initialized from a uniform distribution ∼U(−1,1) and truncated to the range of [−1, 1] (this truncation was enforced throughout training), except for any lateral synapses, which were initialized via ∼U(0,1) and always truncated to the values in the range of [0,1]. All SNN models further used a spiking classifier of the same design—aggregating the spike vector outputs across all layers—as the one proposed for our CSDP SNN system to ensure a fair comparison. In general, we found that these hidden-to-output synapses improved generalization performance across the board. All models/baselines were trained for a fixed 30 epochs for each database.

**Table 1.** Generalization of SNNs trained under different bioplausible schemes. Measurements of generalization accuracy (ACC, in terms of %) of spiking networks trained with different credit assignment processes (means and SDs reported for 10 trials). “Imp.” denotes implementation, whereas the label Sm indicates a small CSDP model and the label Lg indicates a large CSDP model. BP-FFN is the rate-coded comparison model (a backprop-trained feedforward neural network). Number of plastic synapses (NoPS) and total number of synapses (NoS) are also reported for each model (these counts include all classification parameters). Note that training the spiking RBM model requires shrinking the MNIST inputs to a shape of 22 × 22 pixels (*55*). Note that both the unsupervised (“Unsup”) and supervised (“Sup”) CSDP models were each equipped with separate, local classification synapses that were trained alongside and simultaneously with the rest of their parameters, allowing both model variants to readily make class predictions at any point in time (see the “Spike-driven classification and its fast approximation” section for details).

	MNIST ACC (%)	K-MNIST ACC (%)	NoPS	NoS
BP-FNN (Imp.)	98.70 ± 0.02	93.66 ± 0.07	54,572,010	54,572,010
Spiking-RBM (*55*)	89.00	–	250,368	250,368
ML H-SNN (*56*)	91.64	–	3,136	3,136
SNN-LM (*57*)	94.07	–	1,254,400	1,254,400
Power-Law STDP (*58*)	95.00	–	5,017,600	86,937,600
STDP-SNN (*43*) (Imp.)	96.05 ± 0.15	73.82 ± 0.18	7,840,000	207,840,000
DRTP (*39*) (Imp.)	93.55 ± 0.17	78.68 ± 0.13	54,572,010	54,712,010
BFA (*40*) (Imp.)	96.65 ± 0.10	88.75 ± 0.05	54,572,010	54,712,010
L2-SigProp (*34*) (Imp.)	88.58 ± 0.02	70.94 ± 0.04	201,726,010	201,726,010
Loc-Pred (*41*) (Imp.)	93.76 ± 0.03	75.18 ± 0.20	54,782,020	54,782,020
CSDP, Unsup (Ours; Sm)	95.12 ± 0.07	86.55 ± 0.04	7,791,000	7,791,000
CSDP, Unsup (Ours; Lg)	95.96 ± 0.05	89.43 ± 0.16	39,980,000	39,980,000
CSDP, Sup (Ours; Sm)	97.02 ± 0.04	88.49 ± 0.12	7,815,500	7,815,500
CSDP, Sup (Ours; Lg)	97.58 ± 0.05	91.53 ± 0.15	40,040,000	40,040,000

Since all of the neurobiologically plausible credit assignment algorithms, including our own, could support minibatch calculations, we trained all models using minibatches of 500 patterns, randomly sampled without replacement from a training dataset, to speed up simulation. Note that, for CSDP, only one negative sample is created on-the-fly for each positive sensory sample, and for simulation efficiency, all negative samples are appended to the minibatch of original (sensory) samples. Furthermore, the Adam adaptive learning rate (*44*) (with step size η = 0.002) was leveraged to physically adjust synaptic strength values using the updates provided by any one of the simulated algorithms. Each data point/batch was presented to all spiking networks for a stimulus window of *T* = 90 to 150 ms (Δ*t* = 3 ms). Last, note that all spiking networks used the same adaptive threshold update scheme that the CSDP system used—it was observed that this mechanism improved training stability in all cases.

To quantitatively compare models in terms of behavior (i.e., with respect to categorizing the processed visual symbols), we measured, and report in Table 1, the generalization accuracy on unseen (test) samples. Note that accuracy was calculated in the following manner: $\text{acc}=\left(1/N\right)\sum_{n=1}^{N}\left({\text{argmax}}_{c\in C}Y_{n}\equiv {\text{argmax}}_{c\in C}{\bar{Y}}_{n}\right)$ where the matrix **Y** ∈ {0, 1}^*N*×*C*^ contains all of the one-hot encoded labels (**Y***_n_* retrieves/extracts the *n*th row vector of **Y**), while the matrix $\bar{Y}\in R^{N\times C}$ contains all of the collected model predicted class probability vectors (see Materials and Methods for how these probabilities were estimated from the spiking networks). Generalization accuracy values in Table 1 are reported in terms of the mean and SD, i.e., μ ± σ. These statistics were calculated across 10 experimental simulation trials (the random number generator for each trial was seeded with a unique integer). Furthermore, to contextualize the results in terms of biophysical model parameter complexity, we measure and report the number of plastic synapses (NoPS) of each model, for both implemented and prior reported results, as well as the total number of synapses (NoS).

In Table 1, we present the results of our experimental simulations. Notice that, like many biophysical spiking networks, although we do not exactly match the performance of BP-FNNs, our generalization error comes promisingly close. This is despite the fact that every layer of our model is a group of spiking neurons, making inference much noiser than in models that operate with clean continuous rate-coded activities. Furthermore, our CSDP models, particularly the supervised variant CSDP, Sup, comes the closest to matching the BP-FNN rate-coded baseline as compared to other SNN credit assignment procedures. The unsupervised CSDP model only does a bit worse than the supervised one yet effectively operates on par with the STDP-SNN and nearly on par with the BFA-adapted SNN on MNIST, further notably outperforming (both) on K-MNIST. We remark that the BFA SNN offers a competitive baseline—feedback synaptic pathways empirically were found to result in improved overall performance compared to other schemes such as Loc-Pred and L2-SigProp—and, thus, it is promising to observe that CSDP, which does not require any additional feedback connectivity, still effectively outperforms it in terms of generalization ability on test image samples. The STDP-SNN offers a competitive nonfeedback-based baseline on MNIST, although it is a single-layer model [and struggles to work well on deeper architectures, as discussed in (*43*), obtaining only 83.75% on MNIST when applied to a two-hidden layer SNN], yet does not perform well on the harder K-MNIST database. However, on both datasets, the supervised variant of CSDP (both small Sm and Lg models) offers better generalization than the STDP-SNN [even compared to the 96.73% maximal MNIST score reported in (*43*), which required a greedy, layer-by-layer training heuristic scheme], even when CSDP is restricted to using nearly the same number of plastic parameters as the STDP-SNN, i.e., 7 to 8 million plastic synapses. This is despite the fact that the STDP-SNN is able to further enjoy the benefit of using a much larger pool of nonplastic synapses, as revealed by its NoS measurement. In Table 2, we conducted one more experiment to investigate the effect that batch size had on CSDP’s ability to yield effective SNN models, both in terms of classification and sensory input reconstruction ability. We trained the model under different batch size conditions, ranging from size 2 to 500.

**Table 2.** CSDP generalization performance across batch sizes. Measurements of generalization accuracy (ACC, in terms of %; higher is better) and reconstruction binary cross entropy (BCE, in terms of nats; lower is better) on the development sets of MNIST and K-MNIST (means and SDs reported for 10 trials). The SNN model examined for this experiment consisted of 5000 LIFs in the first hidden layer and 1000 LIFs in the second hidden layer. Each batch-size (*B*) variant model was adapted over a stream of 1.5 × 10^6^ samples (30 epochs worth of pattern data) for MNIST and K-MNIST. Note that both the unsupervised (Unsup) and supervised (Sup) CSDP models were each equipped with separate, local classification synapses that were trained alongside and simultaneously with the rest of their parameters, allowing both model variants to readily make class predictions at any point in time (see the “Spike-driven classification and its fast approximation” section for details).

	Batch Size	Supervised		Unsupervised	
		ACC (%)	BCE (nats)	ACC (%)	BCE (nats)
MNIST	*B* = 2	95.19 ± 0.13	143.60 ± 0.24	94.87 ± 0.07	131.74 ± 1.21
	*B* = 20	96.55 ± 0.11	138.28 ± 0.40	95.18 ± 0.22	127.97 ± 0.33
	*B* = 50	96.84 ± 0.14	138.02 ± 0.71	95.44 ± 0.25	128.44 ± 0.22
	*B* = 100	97.05 ± 0.08	135.11 ± 1.29	95.62 ± 0.11	126.36 ± 1.91
	*B* = 200	97.16 ± 0.07	135.84 ± 0.43	95.75 ± 0.28	126.61 ± 0.79
	*B* = 500	97.58 ± 0.05	134.23 ± 0.59	95.96 ± 0.05	126.09 ± 0.83
K-MNIST	*B* = 2	86.35 ± 0.22	344.25 ± 0.84	85.92 ± 0.34	286.46 ± 1.36
	*B* = 20	87.02 ± 0.31	331.93 ± 0.92	86.81 ± 0.16	286.30 ± 0.99
	*B* = 50	88.30 ± 0.17	324.14 ± 1.22	87.81 ± 0.21	287.21 ± 0.92
	*B* = 100	90.00 ± 0.13	315.13 ± 0.55	88.76 ± 0.16	282.64 ± 0.61
	*B* = 200	90.44 ± 0.12	313.57 ± 1.32	88.51 ± 0.13	285.76 ± 2.12
	*B* = 500	91.53 ± 0.15	303.36 ± 0.96	89.43 ± 0.16	284.03 ± 0.81

Observe, in Table 2, that there is a small dependency between model performance and batch size (a known issue with many forms of self-supervised learning in general). Decreasing the batch size from 500 toward 2 leads to, in the worst case, a drop in accuracy of a bit more than 2% on MNIST and more than 5% on K-MNIST for the supervised CSDP model, while there was a degradation of about 1% on MNIST and 3.5% on K-MNIST for the unsupervised variant (reconstruction cross-entropy also went up/degraded by several nats).

### Visualizing CSDP-SNN latent distributions

**Fig. 4.**
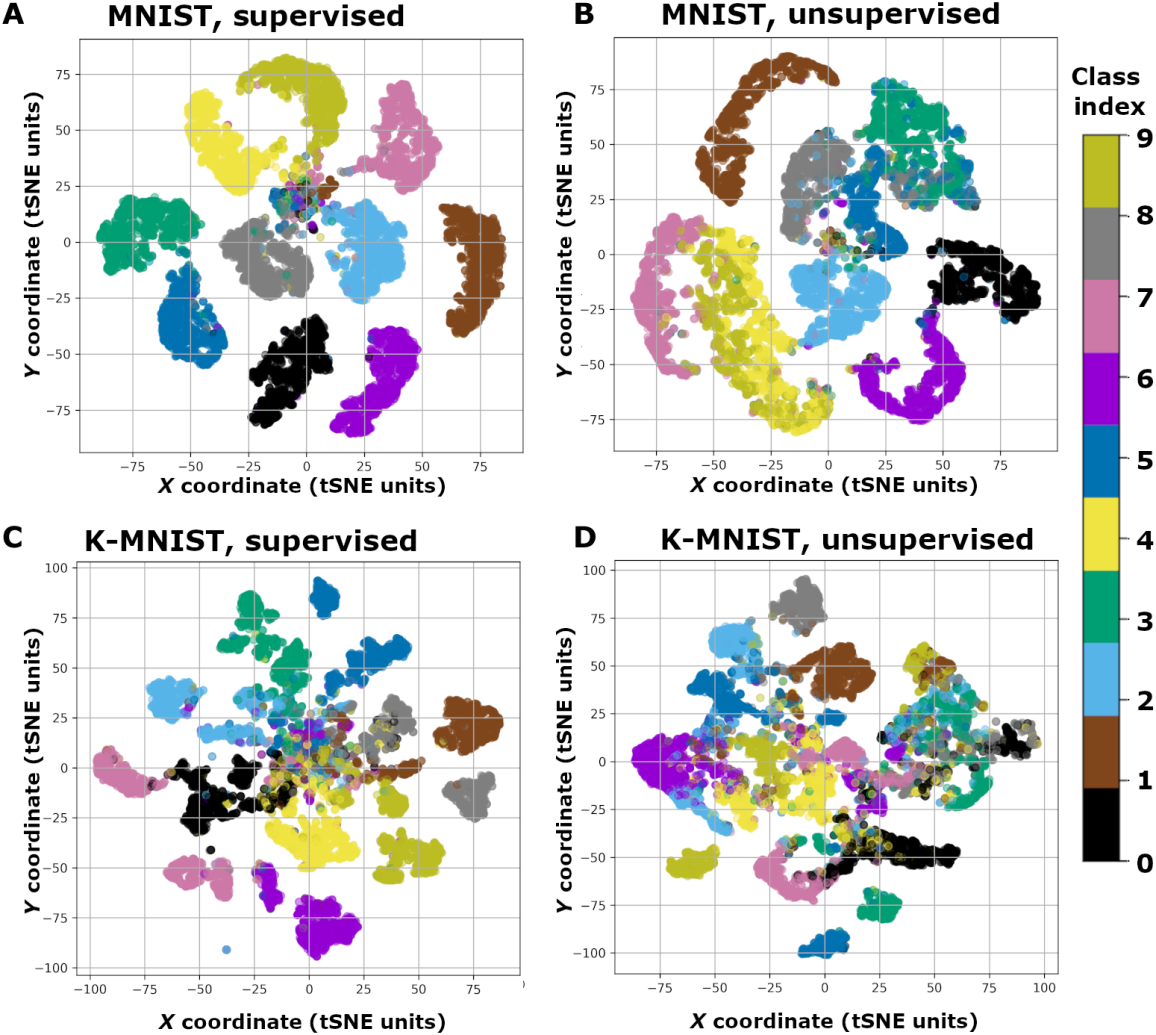
Latent activity patterns acquired by CSDP-trained SNNs. t-SNE visualizations of the latent space induced by recurrent spiking networks learned with the CSDP process. Note that t-SNE coordinate units are technically dimensionless units, and we thus denote them as “tSNE units.” Rate codes are visualized for (**A**) CSDP, Sup (supervised CSDP-trained SNN) on MNIST, (**B**) CSDP, Unsup (unsupervised CSDP-trained SNN) on MNIST, (**C**) CSDP, Sup on K-MNIST, and (**D**) CSDP, Unsup on K-MNIST.

In Fig. 4, we next examined the latent space induced by recurrent spiking circuits trained with CSDP. Specifically, we collected the neural “codes” produced by the top-most hidden layer of each model by converting each temporal spike train produced (in response to a single pattern) to a single rate-code vector **c**. For each data point, we calculate an approximate real-valued, rate-code vector from the circuit’s top layer spike train, i.e., ${\left\{s^{L}\left(t\right)\right\}}_{t=1}^{T}$, as follows

$$c=\left(\gamma_{c}/T\right)\sum_{t}^{T}s^{L}\left(t\right)$$ (12)

with γ*_c_* = 1. After feeding each data point **x** (further omitting its target class input **y** if one is available) in the test set to each model and collecting an approximate top-layer rate-code vector **c**, we visualized the emergent islands of rate codes using t-distributed stochastic neighbor embedding (t-SNE) (*45*). Notice how, for both MNIST (Fig. 4, A and B) and K-MNIST (Fig. 4, C and D), clusters related to each category form, qualitatively demonstrating why CSDP-adapted SNNs are able to classify unseen test patterns effectively. This is particularly impressive for K-MNIST, which is arguably the more difficult of the two datasets, which means that CSDP adaptation is capable of extracting class-centric information from more complicated patterns. Last, observe that the clusters are less separated/distinct in the case of CSDP, Unsup, i.e., Fig. 4 (B and D), as opposed to CSDP, Sup (which is due to the lack of target class mediation).

### Pattern reconstruction and receptive fields

**Fig. 5.**
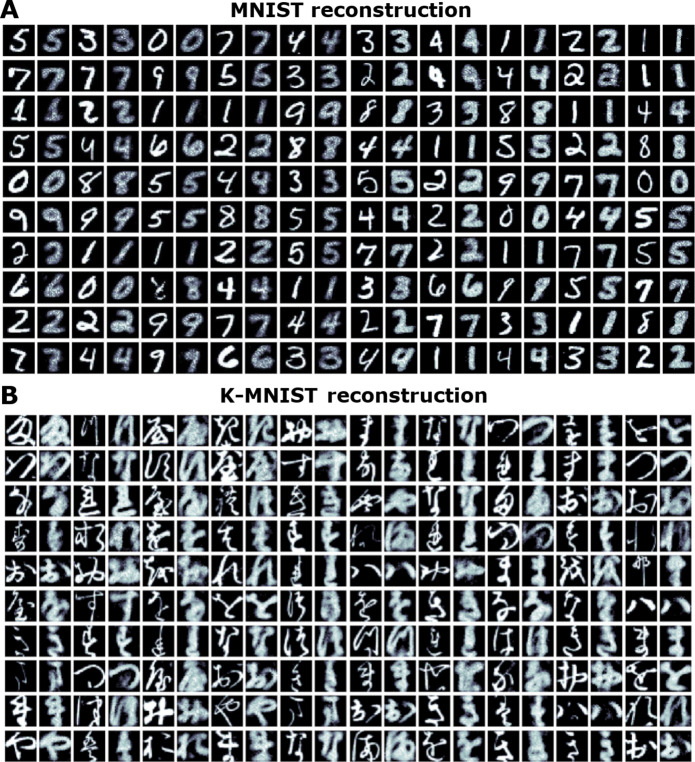
Reconstruction ability of a CSDP-trained SNN. Reconstructed samples produced by the SNN when learned with CSDP. The top row (**A**) presents intercalated original images taken from the MNIST database (randomly sampled) with CSDP model reconstructed images; columns alternate between original and reconstruction patterns (starting with original images in the leftmost column). The bottom row (**B**) presents original images and reconstructed values for the K-MNIST database [formatted in the same way as subfigure (A)].

In Fig. 5, we visualize the pattern reconstruction ability of a CSDP-adapted spiking model (focusing on the CSDP, Sup variant) on randomly sampled, without replacement, image patterns from the MNIST and K-MNIST databases. Desirably, we see that the CSDP model is able to produce reasonable reconstructions of the image patterns, demonstrating that it is able to encode, in its local generative synapses, information needed to decode the sparse spike train representations stored in its internal layers back into sensory input space.

**Fig. 6.**
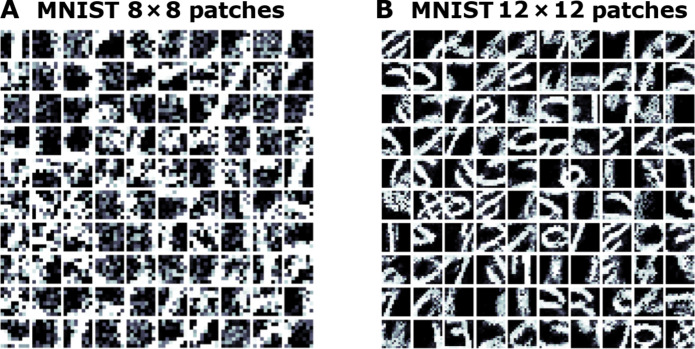
Bottom-layer receptive fields acquired by patch-level CSDP-trained SNNs. The receptive fields of 100 randomly chosen LIF neuronal units, in layer *ℓ* = 1, of unsupervised CSDP models, trained on trained on MNIST sample patches. Shown are the receptive fields for (**A**) 8 × 8 patches and (**B**) 12 × 12 patches.

We lastly investigate the representation capability of our spiking system in the context of image patches. Specifically, we extracted patches of 8 × 8 and 12 × 12 pixels from a subset of the MNIST database (1000 image patterns, 10 from each class) and simulated a CSDP-learned model with two layers of 2000 leaky integrator NPUs each. The learned receptive fields, for each of the two patch settings, of the bottom-most layer of the trained model, i.e., layer *ℓ* = 1, are shown in Fig. 6. Receptive fields in the bottom layer were extracted for visualization by randomly sampling, without replacement, 100 slices of the synaptic matrix **W**^1^. As observed in Fig. 6, the receptive fields extract basic patterns at different resolutions. In the smaller 8 × 8 patch model, we see simpler patterns emerge, such as edges or stroke pieces (“strokelets”) of different orientations. In the larger 12 × 12 patch model, we observe portions of patterns that appear as “mini-templates” that could be used to compose complete digit symbols.

## DISCUSSION

In this study, the proposed process of CSDP offers a powerful yet simple neurobiologically motivated scheme for local self-supervised learning in spiking neural systems. A central finding of our experiments is that iterative (contrastive) learning in a recurrent spiking neuronal circuit with fully parallel neural layer dynamics is possible. This critically resolves the core locality and locking problems, which are also sources of biological implausibility, that plague inference and credit assignment in many models of SNNs, which often rely on forward transmission/inference neuronal circuitry similar to the architectures of modern-day deep neural networks. An important implication of this result is that our framework could be used to guide the effective design of parallel neuromorphic hardware arrangements with asynchronous updating of spiking neuronal dynamics. Furthermore, our results demonstrate that CSDP adaptation successfully learns temporal representations that facilitate effective classification as well as reconstruction of visual sensory inputs—both unsupervised and supervised variants of our circuitry result in spiking neuronal activities that facilitate generative and discriminative capabilities. Notably, CSDP is a viable candidate for instantiation in memristive platforms (*11*, *12*), given that it only requires presynaptic spike and postsynaptic spike/trace information (for how a CSDP circuit might be instantiated on a crossbar, see Supplementary Text). Given that CSDP can be decomposed into a modulatory signal and a presynaptic STDP term (see Supplementary Text), one could take advantage of the long-standing work in emulating STDP and integrate-and-fire functionality through conventional complementary metal-oxide semiconductor technology, possibly using 1-bit analog-digital converters, e.g., oversampled Delta-Sigma modulator units, in crafting CSDP-adapted NPUs.

From a computational neuroscience point of view, our results suggest that a self-supervised contrastive signal could play an important role in local plasticity. This is noteworthy given that it is not guaranteed that feedback pathways or dopamine reward signals are always available to every region of neurons in the brain at every point in time. CSDP demonstrates how synaptic change might be carried out without the presence of feedback synaptic pathways to produce modulatory signals/perturbations or to pass along error mismatch messages [as in spiking predictive coding schemes (*26*, *27*)]. This could prove useful in modeling adaptivity in neuronal circuitry without the presence of globally broadcasted neuromodulation signals based on error or reward values, as in multifactor Hebbian plasticity–based modeling frameworks (*46*, *47*). Furthermore, CSDP strongly complements prior work on neuromimetic models based on contrastive Hebbian learning (*48*), offering a decoupled and nonconditioned form of positive (plus) and negative (minus) phases of plasticity, facilitating the creation of spike-level contrastive credit assignment that could generalize schemes that focus on emulating biological wake phase learning and nonrapid eye movement (NREM) sleep–induced replay (*49*). CSDP fundamentally operates on information locally available to synapses, in an architecture of fully in-parallel operating neuronal layers without requiring differentiable neural activities [which desirably side-steps the need for surrogate functions, which are required by schemes such as backpropagation applied to spiking networks (*21*)], providing a viable candidate inference and learning scheme for thermodynamically efficient processing-in-memory and the instantiation of neuromorphic mortal computers.

### On limitations

Although our online contrastive forward-only credit assignment process for training recurrent, layer-wise parallel SNNs is promising, there are several limitations to consider. First, there are multiple hyperparameter constants to set/configure, and while CSDP models were found to be reasonably robust to most of these in this work, some consideration of the goodness threshold, the learning rate, and the synaptic decay factor should be given in practice (see the Supplementary Materials for further discussion). Next, although the spiking model that we designed operates with bounded synaptic values (between −1 and 1), it does not operate with strictly positive values, which is an important hallmark of neurobiology—we cannot have negative synaptic efficacies. This is an unfortunate limitation that is made worse by the fact that the signs of the synaptic values could change throughout the course of simulated learning. Ultimately, this is in violation of Dale’s Law (*50*) as synapses emanating from the same neuron should all be excitatory or all inhibitory and not a mix. Nevertheless, we remark that this issue could be potentially rectified by constraining all synapses to [0,1], and then introducing an additional set of spiking inhibitory (or inter-inhibitory) neurons coupled to each layer which would provide the inhibitory/depression signals generally offered by negative synaptic strengths. The proportion of inhibitory neurons to excitatory ones could then further be desirably configured to adhere to the ratios of excitatory to inhibitory neurons as reported in work in neurobiology and/or further to maintain a form of excitatory-inhibitory balance. Future work will explore this possible reformulation, as the countering pressure offered by negative synapses is important for a goodness-based contrastive objective such as CSDP to work optimally—ideally, inhibitory neurons should provide the required type of counter-pressure.

Last, we remark that, while CSDP performed well on the datasets that we investigated, the performance gain(s) observed when going from the smaller (Sm, with a bit over 7 million plastic synapses) to larger models (Lg, with a bit over 40 million synapses) was rather modest, particularly on MNIST with less than a percentage point increase (in contrast, on K-MNIST, there was more than a 3% increase when increasing model size/complexity). This possibly indicates that the additional capacity afforded by the extra synaptic parameters is not being as effectively used by the learning process as it could be. In the supplement, further evidence that supports this possibility is provided by an architecture size experiment that we conducted—a law of diminishing returns effect was observed as the model was scaled to larger sizes/capacities (saturating to about 97.67% test accuracy on MNIST for the biggest model). Potential future improvements to CSDP-based model generalization, particularly on image-based sensory inputs, would likely come from the design and integration of useful inductive biases, e.g., convolution or locally connected synaptic structures that approximate convolution rather than just allocating a greater model capacity.

Beyond its use of synapses without a sign constraint, our current recurrent SNN does not include refractory periods, only implementing an instantaneous form of depolarization for all NPUs. However, as mentioned earlier in this article, this could be easily corrected by modifying our spike output functions to include an absolute refractory period, as in (*27*), as well as a relative refractory period. Furthermore, the adaptive thresholds that we presented in the main paper are calculated as a function of the total number of spikes across a layer *ℓ*, at time *t*, whereas it would be more biophysically realistic to have each leaky integrator unit within a layer adapt its own specific scalar threshold. Note that a per-neuron adaptive threshold could be modeled by another ordinary differential equation.

With respect to the CSDP process itself, while we were able to desirably craft a simple update rule that operated at the spike level, there is still the drawback that the rule, much like its rated-coded sources of inspiration (*1*, *2*), requires positive and negative data samples to compute meaningful synaptic adjustments. In this study, we made use of two simple approaches to synthesize negative samples: (i) In the case of the supervised model variant, we exploited the fact that labels were readily available to serve as top-down target class signals which made synthesizing negative samples easy—all we needed to do was simply select one of the incorrect class labels to create a negative target class, or (ii) in the case of the unsupervised model variant, we generated out-of-distribution patterns by interpolating between distinct pattern pairs within a minibatch in tandem with randomly applied (image) rotation. Although our approaches for synthesizing negative data samples are simple and performed on-the-fly, investigating alternative, more sophisticated schemes would prove fruitful. One promising way to do this could be through the use of the predictive/generative synapses of the CSDP spike model we introduced earlier. In other words, the system could dynamically produce data confabulations that would serve as on-the-fly negative patterns. However, the greatest difficulty in synthesizing negative samples via the generative circuitry of our spiking model would be in crafting a process by which ancestral sampling could be efficiently conducted. We remark that crafting an ancestral sampling process in the context of spike trains is not nearly as clear as it is in the realm of rate-coded models, such as in (*2*). Likely, one would need to develop a sampling scheme that is temporal in nature, which might be challenging to formulate in an online, efficient manner.

For the supervised variant of our model, an alternative to synthesizing negative samples would be to design another neural circuit that produces (both positive and negative variations of) a context vector in place of **y** instead of using a provided label—a direction that is also more biologically realistic. Another drawback of using positive and negative samples in the kind of contrastive learning that we do in this work is that both types of data are used simultaneously. In (*1*, *2*), it has been discussed that it will be important to examine alternative schedules for when negative samples are presented to and used by a neural system to adjust its synapses. Another notable challenge facing CSDP, as well as other forms of biological credit assignment, is that of scaling. While this was not explored in this study, CSDP would be amenable to the use of operations such as convolution/deconvolution as well as operate in the context of architectures of depth greater than the four-layer models investigated in this work. Given that CSDP updates only require incoming (presynaptic spikes) information and outgoing values (e.g., postsynaptic information and a cross-layer modulatory signal), using operations useful for processing more complex sensory inputs, e.g., natural images, should be possible. One key difficulty in scaling CSDP, particularly the unsupervised variant, will be in the design of useful negative samples, which will likely be task or data type dependent (this work took advantage of the fact that the inputs were pixel images and were thus amenable to image/pixel transformations; other forms of data would require other means of generating negative data). It would be fruitful for future work to investigate CSDP’s efficacy in adapting more complex spiking neuronal architectures (as well as other types of spiking neuronal dynamics, e.g., as in Izhikevich cells) to a wider variety of problems contexts.

Last, although CSDP results in synaptic updates that are local in terms of neural circuit structure (updates for each layer’s synaptic bundles happen in parallel), the contrastive modulator δ^*ℓ*^(*t*) central to CSDP is spatially nonlocal in and of itself. In other words, it requires activity trace information taken across a layer/group of neurons since the goodness probability is computed via summation across trace values. Biologically, a form of this modulatory signal could come from astrocytic cellular support (*51*, *52*). This would mean that each value within δ^*ℓ*^(*t*), i.e., $\delta_{i}^{\ell }\left(t\right)$ for any neuron *i* in layer *ℓ*, is produced via a “helper cell” that can quickly aggregate readily available information across a group of spatially nearby spiking neuronal cells, embodying the production of transporters for various neurotransmitters (*51*) or serving as the impetus for an increase in long-term potentiation (*52*). In the supplement, we provide one possible neuromorphic implementation of this supporting astrocytic computation.

## MATERIALS AND METHODS

### Datasets used

The data used in for the simulations conducted in this study came from the MNIST and K-MNIST databases. The MNIST dataset (*53*) specifically contains images of handwritten digits across 10 different categories. K-MNIST is a challenging drop-in replacement for MNIST, containing images depicting hand-drawn Japanese Kanji characters (*54*). Each class in this database corresponds to the character’s modern hiragana counterpart, with 10 total classes. For both datasets, image patterns were normalized to the range of [0,1] by dividing pixel values by 255. The resulting pixel “probabilities” were then used to create sensory input spike trains by treating the normalized vector **x**/255 as the parameters for a multivariate Bernoulli distribution. The resulting distribution was sampled at each time step *t* over the stimulus window of length *T*. Note that we did not preprocess the image data any further unlike previous efforts related to spiking networks. However, we remark that it might be possible to obtain better performance by whitening image patterns (particularly in the case of natural images) or applying a transformation that mimics the result of neural encoding populations based on Gaussian receptive fields. From both MNIST and K-MNIST, which each contained 60,000 training samples (and a test set of 10,000 samples), a validation/development subset of 10,000 patterns (1000 from each class) was created by randomly sampling without replacement from each database’s training set (the development subset was used to manually tune/select hyperparameter values). These subsets were used to aid in selecting/verifying constants/hyperparameters.

### Spiking model specification details

All of the synapses of the full model, composed of *L* layers of NPUs, can be represented as a set of matrices contained in the synaptic parameter construct Θ = {**W**^1^, **V**^1^, **M**^1^, **B**^1^, …, **W**^*ℓ*^, **V**^*ℓ*^, **M**^*ℓ*^, **B**^*ℓ*^, …**W***^L^*, **M***^L^*, **B***^L^*} (the top layer *L* does not contain any top-down recurrent synapses as there would be no layer above *L*). Matrix **W**^*ℓ*^ ∈ [−1, 1]^*J*_*ℓ*_×*J*_*ℓ*−1_^ contains the local bottom-up synaptic connections, **V**^*ℓ*^ ∈ [−1, 1]^*J*_*ℓ*_×*J*_*ℓ*+1_^ contains the local top-down recurrent synapses, **M**^*ℓ*^ ∈ [0, 1]^*J*_*ℓ*_×*J*_*ℓ*_^ holds the lateral inhibition synapses, and the **B**^*ℓ*^ ∈ [−1, 1]^*J*_*ℓ*_×*C*^ stores the optional target class-mediating connections. The values of all synaptic strengths in our model are constrained to remain in the range [−1, 1] except for the lateral ones, which are constrained to nonnegative values [0,1].

In accordance with our CSDP scheme, the Hebbian-like adjustment rules for all possible synaptic connections that project to layer *ℓ* are specifically

$$\delta_{i}^{\ell }\left(t\right)=\partial C\left[z^{\ell }\left(t\right),y_{\text{type}}\right]/\partial z_{i}^{\ell }\left(t\right)$$ (13)

$$\Delta W_{\mathrm{ij}}^{\ell }=\left[R_{E}\delta_{i}^{\ell }\left(t\right)s_{j}^{\ell -1}\left(t-\Delta t\right)\right]+\lambda_{d}\left\{s_{i}^{\ell }\left(t\right)\left[1-s_{j}^{\ell -1}\left(t-\Delta t\right)\right]\right\}$$ (14)

$$\Delta V_{\mathrm{ij}}^{\ell }=\left[R_{E}\delta_{i}^{\ell }\left(t\right)s_{j}^{\ell +1}\left(t-\Delta t\right)\right]+\lambda_{d}\left\{s_{i}^{\ell }\left(t\right)\left[1-s_{j}^{\ell +1}\left(t-\Delta t\right)\right]\right\}$$ (15)

$$\Delta M_{\mathrm{ij}}^{\ell }=\left[R_{I}\delta_{i}^{\ell }\left(t\right)s_{j}^{\ell }\left(t-\Delta t\right)\right]+\lambda_{d}\left\{s_{i}^{\ell }\left(t\right)\left[1-s_{j}^{\ell }\left(t-\Delta t\right)\right]\right\}$$ (16)

$$\Delta B_{\mathrm{ij}}^{\ell }=\left[R_{E}\delta_{i}^{\ell }\left(t\right)s_{y,j}^{\;}\left(t-\Delta t\right)\right]+\lambda_{d}\left\{s_{i}^{\ell }\left(t\right)\left[1-s_{y,j}\left(t-\Delta t\right)\right]\right\}$$ (17)

In this study, we set the decay factor for the second term of our local adjustment rule to be λ_d_ = 0.00005 (typically smaller values were found to result in the best performance). In the Supplementary Materials, we provide a pseudocode that fully depicts the mechanics of the inference and learning underlying a CSDP-trained spiking circuit.

### A CSDP-SNN as a spiking generative model

To incorporate reconstruction/generative potential into our SNN model, we generalize the local generation scheme of the predictive forward-forward (PFF) scheme (*2*) and introduce one more set of matrices which contain the generative synapses—this means that we extend our model parameters to Θ ∪ Θ*_g_* where $\Theta_{g}={\left\{G^{\ell }\right\}}_{\ell =1}^{L}$. Desirably, each synaptic matrix **G**^*ℓ*^∈ [−1, 1]^*J*_*ℓ*_×*J*_*ℓ*+1_^ is directly wired to each layer *ℓ*, forming a local prediction of activity **s**^*ℓ*^(*t*) as follows

$$v_{\mu }^{\ell }\left(t+\Delta t\right)=v_{\mu }^{\ell }\left(t\right)+\left(\Delta t/\tau_{m}\right)\left\{-v_{\mu }^{\ell }\left(t\right)+R_{E}\left[G^{\ell +1}\cdot s^{\ell +1}\left(t\right)\right]\right\}$$ (18)

$$s_{\mu }^{\ell }\left(t\right)=v_{\mu }^{\ell }\left(t+\Delta t\right)>v_{\text{thr},\mu }^{\ell },v_{\mu }^{\ell }\left(t\right)=v_{\mu }^{\ell }\left(t+\Delta t\right)⊙\left[1-{s_{\mu }}^{\ell }\left(t\right)\right]$$ (19)

$$e^{\ell }\left(t\right)=s_{\mu }^{\ell }\left(t\right)-s^{\ell }\left(t\right)$$ (20)

where ⊙ denotes elementwise multiplication and ⋅ denotes matrix-vector multiplication. We observe that error mismatch units **e**^*ℓ*^(*t*) have been introduced that specialize in tracking the disparity between prediction spikes $s_{\mu }^{\ell }\left(t\right)$ and a current representation spikes **s**^*ℓ*^(*t*). To update each generative matrix **G**^*ℓ*^, we may then use the simple Hebbian update rule

$$\Delta G^{\ell }=\left[R_{E}e^{\ell -1}\left(t\right)\right]\cdot {\left[s^{\ell }\left(t\right)\right]}^{T}$$ (21)

where [**s**^*ℓ*^(*t*)]*^T^* indicates that the transpose operator is applied to **s**^*ℓ*^(*t*). When making local predictions and calculating mismatches as above, the full spiking neural system’s optimization objective, at time *t*, can be written down as

$$F\left(t,\Theta \right)=\sum_{\ell =0}^{L}\left\{ \begin{array}{cc} C\left[z^{\ell }\left(t\right),y_{\text{type}}\right] & \text{if}\;\ell =L\\ \left(1/2\right)∥s_{\mu }^{\ell }\left(t\right)-s^{\ell }\left(t\right){∥}_{2}^{2} & \text{if}\;\ell =0\\ C\left[z^{\ell }\left(t\right),y_{\text{type}}\right]+\left(1/2\right)∥s_{\mu }^{\ell }\left(t\right)-s^{\ell }\left(t\right){∥}_{2}^{2} & \text{otherwise}. \end{array}\right.$$ (22)

Notice that each layer of the system is maximizing its goodness while further learning a (temporal) mapping between its own spike train to the one of the layer below it (i.e., a mapping that minimizes a measurement of local predictive spike error).

The mismatch units we model explicitly in Eq. 20 directly follow from taking the partial derivative of the local objective in Eq. 22 with respect to $s_{\mu }^{\ell }\left(t\right)$. Last, across a full stimulus window of length *T*, the global objective that our spiking system would be optimizing is the following sequence loss

$$F\left(\Theta \right)=\sum_{t=1}^{T}F\left(t,\Theta \right)$$ (23)

**Fig. 7.**
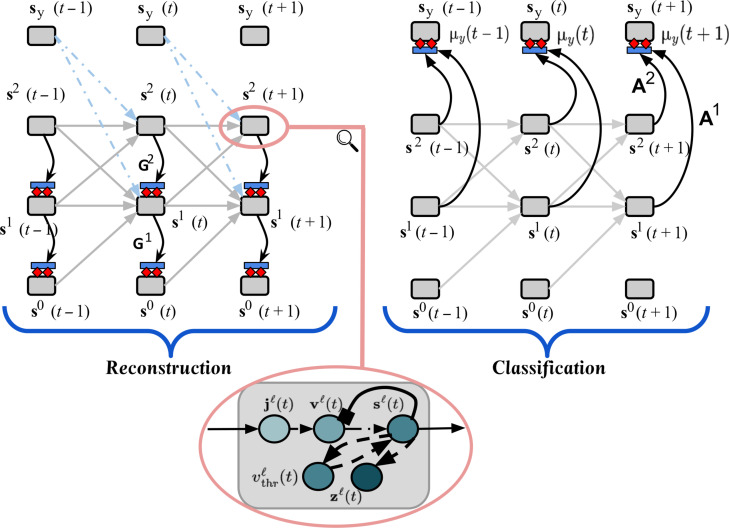
Reconstruction and classification processes of a CSDP-trained SNN. An overview of the task-specific extensions made to the CSDP-adapted recurrent spiking network, unfolded over three time steps, which processes a sensory spike train {**s**^0^(0), …, **s**^0^(*t*), …, **s**^0^(*T*)}) with possibly an available corresponding target class spike train {**s***_y_*(0), …, **s***_y_*(*t*), …, **s***_y_*(*T*)}). The zoomed-in inset depicts, internally, that each activation layer is made up of at least four components, i.e., an electrical current model **j**^*ℓ*^(*t*), a voltage model **v**^*ℓ*^(*t*), a spike response function **s**^*ℓ*^(*t*), and an adaptive threshold $v_{\text{thr}}^{\ell }\left(t\right)$. The leftmost unfolded model (reconstruction) introduces additional synaptic bundles **G**^1^ and **G**^2^ that allow a layer *ℓ* to make local predictions of the neuronal activities of layer *ℓ* − 1. The rightmost unfolded model (classification) introduces extra synaptic connections **A**^1^ and **A**^2^ that allow the layers to make a prediction μ*_y_*(*t*) of the target class spikes **s***_y_*(*t*) at time *t*. Note that the light dot-dashed arrows, representing the class-modulating synaptic connections, are not used in the case of the unsupervised model’s reconstruction process (since class context is not available for this case).

In effect, the spiking neural system that we simulate in this work attempts to incrementally optimize the sequence loss in Eq. 23 when processing a data sample (**x**, **y**) (or **x** in the case of unsupervised learning setups), conducting a form of online adaptation by computing synaptic adjustments given the current state of Θ at time *t*. In Fig. 7A, we depict how the local (spike) predictions are made in our recurrent spiking architecture.

### Pattern reconstruction details

For a given CSDP-trained SNN, a single reconstructed pattern $\hat{x}$ was created by computing an average across (clipped) activation traces of the bottom-most predictor’s output spikes, collected during the *T*-length stimulus window as follows

$$\begin{array}{c} \hat{x}=\left(1/T\right)\sum_{t=1}^{T}z_{\mu }^{0}\left(t\right),\\ \text{where}\;{z_{\mu }}^{0}\left(t\right)=\left\{{z_{\mu }}^{0}+\left(\Delta t/\tau_{tr}\right)\left[-{z_{\mu }}^{0}\left(t\right)\right]\right\}⊙\left[1-{s_{\mu }}^{0}\left(t\right)\right]+{s_{\mu }}^{0}\left(t\right) \end{array}$$ (24)

where $z_{\mu }^{0}\left(t\right)$ is the activation trace of the output prediction spike vector $s_{\mu }^{0}\left(t\right)$, produced by the local prediction of layer *ℓ* = 0 from layer *ℓ* = 1 (see Eq. 18), at simulation step *t*.

Note that for the reconstruction analysis, the class **y** was not clamped in our model for reconstruction to be evaluated. This means that the model could only use class-specific information it had previously stored in its synapses to determine how to best reconstruct test samples.

### Spike-driven classification and its fast approximation

To perform classification with the recurrent spiking system model developed over the previous few sections, one must run a scheme similar to (*1*, *2*) where one iterates over all possible class values, i.e., setting the target vector **y** equal to the one-hot encoding of each class *c* ∈ {1, 2, …, *C*}. Specifically, for each possible class index *c*, we create a candidate target/class vector **y** = 1*_c_*. 1*_c_* is the indicator function, returning one when the index *i* (of the label space) corresponds to the correct class index *c* ∈ {1, 2, …, *C*} and zero otherwise. We then present **y** and the sensory input **x** to the system over a stimulus window of *T* steps and record the goodness values summed across layers, averaged over time, i.e., $G_{y=c}=\left(1/T\right)\sum_{t=1}^{T}\sum_{\ell =1}^{L}\sum_{j=1}^{J_{\ell }}{\left[{z_{j}}^{\ell }\left(t\right)\right]}^{2}$. This time-averaged goodness value is computed for all class indices *y* = 1, 2, …, *C*, resulting in an array of *C* scores, i.e., $G=\left\{G_{y=1},G_{y=2},\ldots ,G_{y=C}\right\}$. The argmax operation is applied to this array *G* to obtain the index of the class with the highest average (aggregate) goodness value, i.e., *c*_pred_ = argmax_*c*∈*C*_*G*.

While the above per-class classification process would work fine for reasonable values of *C*, it would not scale well to a high number of classes, i.e., very large values of *C*, given that the above process requires simulating the spiking network over *C* stimulus windows to compute a predicted label index that corresponds to maximum goodness. To circumvent this expensive form of classification, we constructed a simple modification which circumvents the need to conduct this classification process entirely by integrating a simple spiking classification subcircuit to the recurrent spiking system. This classifier, which takes in as input the spike output produced at the top level of the recurrent system, i.e., **s***^L^*(*t*), is jointly learned with the rest of the model parameters. This merely entails extending Θ one more time so as to include classification-specific synapses, i.e., Θ ∪ Θ*_c_* where $\Theta_{c}={\left\{A^{\ell }\right\}}_{\ell =1}^{L}$. Concretely, the classifier module operates according to the following dynamics

$$v_{y}\left(t+\Delta t\right)=v_{y}\left(t\right)+\left(\Delta t/\tau_{m}\right)\left\{-v_{y}\left(t\right)+R_{E}\left[\sum_{\ell =1}^{L}A^{\ell }\cdot s^{\ell }\left(t\right)\right]\right\}$$ (25)

$$\mu_{y}\left(t\right)=v_{y}\left(t+\Delta t\right)>v_{\text{thr}}^{y},v_{y}\left(t\right)=v_{y}\left(t+\Delta t\right)⊙[1-\mu_{y}\left(t\right)]$$ (26)

$$v_{\text{thr}}^{y}=v_{\text{thr}}^{y}+\lambda_{v}\left[\sum_{j=1}^{C}\;\mu_{y,j}\left(t\right)-1\right]$$ (27)

where we notice, in Eq. 25, that the class prediction voltage **v***_y_*(*t*) is the result of aggregating across spike vector messages transmitted from layers *ℓ* = 1 to *ℓ* = *L*. Each synaptic matrix **A**^*ℓ*^ that makes up the spiking classifier is adjusted according to the following Hebbian rule

$$\Delta A^{\ell }=R_{E}\left[\mu_{y}\left(t\right)-s_{y}\left(t\right)\right]\cdot {\left[s^{\ell }\left(t\right)\right]}^{T}$$ (28)

Note that, in the case of unsupervised CSDP, **s***_y_*(*t*) is not provided as class contextual input (the class-modulating synapses are zeroed out/removed), even if the model is equipped with the above skip-layer classification synapses (i.e., the class spike error signal only affects the plasticity of the classification synapses ${\left\{A^{\ell }\right\}}_{\ell =1}^{L}$). If these additional synaptic classification parameters are learned, then, at test time, when synaptic adjustment would be disabled, one can simply provide the spiking system with the sensory input **x** with no target class **y** and obtain a estimated class output $\bar{y}$. This $\bar{y}$ is a function of the system’s predictions across time, i.e., it is the approximate output distribution computed as $p\left(y|x;\Theta \right)\approx \bar{y}=\text{exp}\left[\sum_{t=1}^{T}\;\mu_{y}\left(t\right)\right]/\sum_{c=1}^{C}\text{exp}{\left[\sum_{t=1}^{T}\;\mu_{y}\left(t\right)\right]}_{c}$. Note that the conditional log likelihood log*p*(**y**∣**x**; Θ) of the spiking model can now be measured using this approximate label probability distribution. Although learning this spiking classifier entails a memory cost of up to *L* additional synaptic matrices, the test-time inference of the spiking system will be substantially faster, thus making it more suitable for on-chip computation. In Fig. 7B, our recurrent spiking neural architecture is shown making predictions of the target class’s spike train across three time steps.
