# Supplementary material for: Contrastive signal–dependent plasticity: Self-supervised learning in spiking neural circuits
Source: Sci Adv. 2024 Oct 23;10(43):eadn6076. doi: 10.1126/sciadv.adn6076 (PMC11639678; doi:10.1126/sciadv.adn6076)
Supplement: Supplementary file 1 — Supplementary Text Fig. S1 Tables S1 to S6 [file sciadv.adn6076_sm.pdf]

Science Advances
AAAS

Supplementary Materials for

Contrastive signal–dependent plasticity: Self-supervised learning in spiking neural circuits

Alexander G. Ororbia

Corresponding author: Alexander G. Ororbia, ago@cs.rit.edu

Sci. Adv. **10**, eadn6076 (2024)
DOI: 10.1126/sciadv.adn6076

This PDF file includes:

Supplementary Text
Fig. S1
Tables S1 to S6

Supplementary Text

Layer Size Experiments and Model Ablations

We conducted an experiment probing the performance of a CSDP model (the supervised variant) with respect to its complexity. Specifically, we simulated the model with different first hidden LIF layer sizes (the first hidden layer size was selected as the driving experimental variable). The second hidden layer was set to approximately be one fifth of the size of the first layer in each case (this was a useful ratio empirically found in preliminary experimentation). The model's generalization/test accuracy (ACC) and (binary) reconstruction cross-entropy (BCE) were recorded and the results of this experiment are reported in table S1. We observe that at least 4000-5000 neurons in the first layer are needed to obtain good performance (with respect to classification on MNIST). The units added beyond this range did provide further improvement (in terms of generalization) though a diminishing returns trend was observed as model complexity reached the maximal layer size studied. BCE also reasonably improves up to about 5000 units in the first layer and effectively levels out (in the range of 131-134 nats) beyond this.

Furthermore, we performed a small ablation test of our CSDP model on MNIST, investigating the value/effect that certain components had on overall model performance (in terms of generalization accuracy and binary reconstruction cross entropy). table S2 examines eight possible ablated configurations related to the CSDP-model: with or without the adaptive threshold, with or without the top-down recurrent synapses, and with or without the class-modulating (skip) synapses. With respect to classification, the results show that all three of these are useful elements in obtaining the best possible performance – with the adaptive threshold being one of the most important elements (its removal leads to a drop of nearly 2%) – since removing all three leads to a low test accuracy of about 95% (as compared to the 97.58% of the full model) and high/poor BCE of 159 nats (as compared to 134 nats of the full model). However, while a consistent, overall performance drop appears to be induced by the removal of these three components, this drop is not a very large nor significant. This indicates that the top-down synapses, the additional class-modulating synapses, and the adaptive threshold do not result in the bulk of the CSDP-SNN's base performance – it is the CSDP-trained feedforward synapses that are responsible for this. We hypothesize that more complex tasks, such as processing time-varying data or learning part-whole hierarchies from sensory input, would better showcase and more strongly demonstrate the value of the top-down recurrent and skip class/context-modulating synapses (the integration of these elements were motivated to provide upper-layer constraints or a top-down bias on the lower layers of neuronal activity as the message passing is carried out across the SNN layers during its iterative processing of the input). This is an aspect of the SNN model architecture, and its relation to more complex tasks and data streams, that we will explore in future work.

Operators and Model Variables / Constants

In tables S3 and S4, we collect and briefly define several acronyms, abbreviations, key symbols, and operators used throughout the paper. In table S5, we list and describe the key variables for CSDP as well as indicating if they are plastic or not. Finally, in table S6, we list and describe CSDP model constants. Note that model constants were lightly tuned using a development/validation (held-out) subset of MNIST/K-MNIST of 10000 samples (randomly sampled without replacement from the original $60,000$ sample training set of a given database). The development subset(s) was (were) used to manually tune/select hyperparameter values.

Hyperparameter Sensitivity. Models are expected to be robust to most hyperparameter values with respect to the neuronal dynamics, provided that they are chosen in reasonable ranges – such as the sparsity constraint value for the adaptive threshold (which should not be too high to prevent thresholds from jumping up too quickly) and the base voltage threshold (which should not be too high in terms of decivolts so as to reduce the likelihood of spikes). In terms of the plasticity/learning dynamics, the models are expected to be most sensitive to the goodness threshold θ_z – we found that somewhat higher values, e.g., $\theta_z = 10$, were more effective.

One insight uncovered through preliminary experimentation was that it was important to select the values for the learning rate η_w and the synaptic decay factor λ_d in terms of the learning setting "scale" at which the synaptic updates were occurring. This meant that for larger batch sizes, a higher learning rate (e.g., 0.002) and a lower decay yielded more effective/robust performance, whereas for smaller batch sizes (approaching online updates with $1 - 2$ samples), a lower learning rate with a higher decay was more useful/appropriate. Heuristically, we found that, for MNIST and K-MNIST, a ratio of $\lambda_d/\eta_w = 4$ between the decay rate and learning rate proved to be a useful heuristic (meaning that the modeler would only need to select η_w if this heuristic ratio is employed).

Deriving CSDP from the Goodness Contrastive Functional

Here, we show how to derive a simplified form of CSDP from the goodness contrastive functional we presented in the main article for the case of using spikes directly. Computing goodness with emitted neuronal spikes (omitting t for notational clarity/simplicity) is done in the following manner:

$$p(y_{type} = 1; \mathbf{s}^\ell) = 1/\Big(1 + \exp\big(-\big[(\Sigma_{k=1}^{J_\ell}(\mathbf{s}_k^\ell)^2) - \theta_z\big]\big)\Big) \tag{1}$$

while the contrastive functional to maximize is:

$$\mathcal{C}(\mathbf{s}^\ell(t), y_{type}) = \Big(y_{type} \log p(y_{type} = 1; \mathbf{s}^\ell) + (1 - y_{type}^\ell) \log p(y_{type} = 0; \mathbf{s}^\ell)\Big). \tag{2}$$

To obtain the update for a synaptic matrix such as $\mathbf{W}^\ell$ (the bottom-up synapses in both the supervised and unsupervised variants of CSDP), we take the derivative of Equation 2 with respect to a single synapse W_{ij}^ℓ as follows:

$$\frac{\partial \mathcal{C}(\mathbf{s}^\ell(t), y_{type})}{\partial W_{ij}^\ell} = \frac{\partial \mathcal{C}(\mathbf{s}^\ell(t), y_{type})}{\partial s_i^\ell(t)} \frac{\partial s_i^\ell(t)}{\partial v_i^\ell(t)} \frac{\partial v_i^\ell(t)}{\partial j_i^\ell(t)} \frac{\partial j_i^\ell(t)}{\partial W_{ij}^\ell}. \tag{3}$$

Going through each local term in the above chain rule expression, and setting $p(y_{type} = 0; \mathbf{s}^\ell) = 1 - p(y_{type} = 1; \mathbf{s}^\ell)$, we obtain the derivative of the functional with respect to the goodness probability itself:

$$\frac{\partial \mathcal{C}(\mathbf{s}^\ell(t), y_{type})}{\partial p(y_{type} = 1; \mathbf{s}^\ell)} = \frac{y_{type}}{p(y_{type} = 1; \mathbf{s}^\ell)} - \frac{(1 - y_{type})}{1 - p(y_{type} = 1; \mathbf{s}^\ell)} \tag{4}$$

followed by the derivative of the goodness probability with respect to the sum of spikes/activities minus the goodness threshold:

$$\frac{\partial p(y_{type} = 1; \mathbf{s}^\ell)}{\partial (\sum_{k=1}^{J_\ell}(s_k^\ell)^2) - \theta_z} = \frac{\exp\big(-[(\sum_{k=1}^{J_\ell}(s_k^\ell)^2) - \theta_z]\big)}{(1 + \exp\big(-[(\sum_{k=1}^{J_\ell}(s_k^\ell)^2) - \theta_z]\big)^2} \tag{5}$$

and finally we obtain the following key local derivatives for post-synaptic neuron i:

$$\frac{\partial(\sum_{k=1}^{J_\ell}(s_k^\ell)^2) - \theta_z}{\partial \mathbf{s}_i^\ell} = 2s_i^\ell, \quad \frac{\partial v_i^\ell}{\partial j_i^\ell} = \frac{\Delta t}{\tau_m} R_m, \quad \frac{\partial j_i^\ell}{\partial W_{ij}^\ell} = s_j^{\ell-1} \tag{6}$$

which are the derivative of the thresholded activity sum with respect to the activities, the derivative of the voltage/membrane potential with respect to the electrical current, and the derivative of the electrical current with respect to the synapse W_{ij}^ℓ that connects pre-synaptic neuron j to post-synaptic neuron i. Finally, we choose to apply the straight-through estimator for the derivative of the spike activity of post-synaptic neuron i with respect to its voltage, i.e., $\partial s_i^\ell / \partial v_i^\ell = 1$ (this does mean a biased estimator of the derivative is being used to make the derivation simpler; we remark that one could alternatively employ a more accurate surrogate function for this derivative as is common in the brain-inspired computing literature).

Given the above local terms, we may rewrite the chain rule equation (Equation 3) and compose the final update rule, for positive samples, in the following manner:

$$\left.\frac{\partial \mathcal{C}(\mathbf{s}^\ell, y_{type})}{\partial W_{ij}^\ell}\right|_{y_{type}=1} = \overbrace{\left(\frac{2R_m\Delta t}{\tau_m}\left(\frac{y_{type}}{p(y_{type}=1;\mathbf{s}^\ell)}\right)\left(\frac{\exp(-\xi)}{(1+\exp(-\xi))^2}\right)\right)}^{\text{Positive Modulator } \delta_{i,\text{pos}}^\ell}\overbrace{\left(s_i^\ell s_j^{\ell-1}\right)}^{\text{Hebbian term}}, \tag{7}$$

$$\left.\frac{\partial \mathcal{C}(\mathbf{s}^\ell, y_{type})}{\partial W_{ij}^\ell}\right|_{y_{type}=1} = \delta_{i,\text{pos}}^\ell \left(s_i^\ell s_j^{\ell-1}\right) \tag{8}$$

where the substitution variable is $\xi = (\sum_{k=1}^{J_\ell}(s_k^\ell)^2) - \theta_z$. For negative samples, the final update rule would be:

$$\left.\frac{\partial \mathcal{C}(\mathbf{s}^\ell, y_{type})}{\partial W_{ij}^\ell}\right|_{y_{type}=0} = \overbrace{-\left(\frac{2R_m\Delta t}{\tau_m}\left(\frac{(1-y_{type})}{1-p(y_{type}=1;\mathbf{s}^\ell)}\right)\left(\frac{\exp(-\xi)}{(1+\exp(-\xi))^2}\right)\right)}^{\text{Negative Modulator } \delta_{i,\text{neg}}^\ell}\overbrace{\left(s_i^\ell s_j^{\ell-1}\right)}^{\text{Hebbian term}} \tag{9}$$

$$\left.\frac{\partial \mathcal{C}(\mathbf{s}^\ell, y_{type})}{\partial W_{ij}^\ell}\right|_{y_{type}=0} = \delta_{i,\text{neg}}^\ell \left(s_i^\ell s_j^{\ell-1}\right). \tag{10}$$

To obtain a descent-based (minimizing) optimization direction, one can multiply the above derived rules (as well as the contrastive functional in Equation 2) by negative one.

Note that the CSDP rule presented in the main paper modifies the above partial derivatives (Equations 8 and 10) a bit further by replacing the post-synaptic spike vector $\mathbf{s}^\ell$ with the activity trace vector $\mathbf{z}^\ell$ (the coefficients $2\Delta t/\tau_m$ are omitted because the scaling is handled by the trace's time constant and the optimization rule's learning rate) and further adding a decay (or an anti-Hebbian) term to the synaptic dynamics (to facilitate long-term synaptic depression). In effect, if we examine the above derived rules in Equations 8 and 10 further, we notice that a CSDP update is effectively a product of two terms – a modulatory signal (all values of which are folded into $\delta^\ell(t)$) and a Hebbian term (the post-synaptic spike times the pre-synaptic spike).

More importantly, if we slightly adjust the above Equations 8 and 10, and place back in the time label t, to adhere to the context of the main paper's formulation, we get:

$$\frac{\partial \mathcal{C}(\mathbf{s}^{\ell}(t), y_{type})}{\partial W^{\ell}_{ij}}\Bigg|_{y_{type}=1} = \overbrace{\delta^{\ell}_{i,\text{pos}}(t)}^{\text{Positive Modulator}} \overbrace{\Bigg(z^{\ell}_{i}(t) s^{\ell-1}_{j}(t - \Delta t) \Bigg)}^{\text{Pre-synaptic-driven STDP term}}, \quad (11)$$

$$\frac{\partial \mathcal{C}(\mathbf{s}^{\ell}(t), y_{type})}{\partial W^{\ell}_{ij}}\Bigg|_{y_{type}=0} = \overbrace{\delta^{\ell}_{i,\text{neg}}(t)}^{\text{Negative Modulator}} \overbrace{\Bigg(z^{\ell}_{i}(t) s^{\ell-1}_{j}(t - \Delta t) \Bigg)}^{\text{Pre-synaptic-driven STDP term}} \quad (12)$$

where we see that CSDP's relationship to spike-timing-dependent plasticity (STDP), specifically the trace-based formulation of STDP. CSDP, at its core, is essentially a switch between two modulated pre-synaptic-driven STDP rules (our use of the descriptor 'pre-synaptic-driven' indicates that the STDP update is triggered by pre-synaptic events or spike pulse occurrences). For positive samples, CSDP applies a positively-modulated pre-synaptic-driven STDP adjustment whereas, for negative samples, CSDP entails application of a negatively-modulated pre-synaptic-driven STDP adjustment.

Finally, note that, in the main paper, the modulation vector signal $\delta^{\ell}(t)$ encapsulates the positive and negative modulation signals multiplied by the post-synaptic trace values, i.e., each value within it can be rewritten as: $\delta^{\ell}_{i}(t) = (\delta^{\ell}_{i,\text{pos}} y_{type} + \delta^{\ell}_{i,\text{neg}} y_{type}) z^{\ell}_{i}(t)$, where the binary label y_{type} turns on only one of the two modulatory signals (given it only takes on binary values).

Alternative Plasticity Scheme - The Gated-Voltage Rule

An alternative form to the trace-centered rule we used in this study simply replaces the left argument of the local loss function as follows: $\mathcal{C}(\mathbf{v}^{\ell}(t) \odot \mathbf{s}^{\ell}(t), y_{type})$. This is what we refer to as the "gated voltage rule" which has the advantage of no longer requiring the tracking of a spike activity trace, i.e., it only requires the use of the spike vector at t as a binary multiplicative gate against the current voltage values. However, our goal was to avoid using voltage directly in the plasticity updates since this would mean that the voltage of neuron i (in layer ℓ) would be used in the synaptic updates for all other neurons in layer ℓ ($\forall k \neq i$), which is biologically implausible. Furthermore, we found that, in preliminary experiments, this rule slightly, albeit consistently, underperformed the trace-based form of the loss and we thus do not investigate it further in this work.

A Note on Using Class Values as Inputs

In the supervised CSDP model of the main paper, we made the modeling choice to represent class labels as a separate input (that gets encoded as a target spike train). This choice of inputting the class is (slightly) different from (*1*), which chose to directly overlay the labels onto the image pixel space. We note that nothing prevents the modeler from doing the same in our framework and this could be done if desired, further saving on a small number of plastic synapses (i.e., $10 \times J_1$ for the first layer of neuronal cells). We particularly chose to separate out the labels from the sensory inputs as we envisioned in future incarnations/generalizations of the CSDP SNN architecture that other types of target inputs could be provided beyond labels, such

as outputs from other neuronal circuits, which would then generally not be directly overlaid over the sensory input.

A Possible Neuromorphic Construction of CSDP Modulation

In the main paper, we highlighted that, neurobiologically, one possible instantiation of the key CSDP modulatory signal $\delta^\ell(t)$ (further treated earlier in this supplement as a gated summation of a positive modulatory value δ^ℓ_{pos} and a negative one δ^ℓ_{neg}), was in the form of astrocytic support. Neuromorphically, implementing this could be done by simply integrating into each layer one single additional "helper" neuronal unit – the astrocyte for layer ℓ. This helper cell's role would be to only sum up the trace values (or spikes, if trace hardware support is not designed) emitted/corresponding to a particular group of neural hardware units using non-plastic memristor synapses, which would connect in a feedforward fashion each neuron in group/layer ℓ to the relevant astrocyte unit. This same astrocyte would also receive one additional external input value of either one or zero (which indicates whether a processed sample was real or confabulation and, as seen earlier in this supplement, switches between CSDP's positive or negative modulated STDP adjustment) and finally wire back to these same neurons in ℓ, also with fixed/non-plastic memristor synapses. This fixed recurrent set of fixed synaptic pathways would be used to deposit CSDP's requisite contrastive modulatory signal to each neuron in the group/layer, i.e., a $\delta^\ell_i(t)$ within $\delta^\ell(t)$ would be deposited/back-transmitted to spiking neuronal cell i in layer ℓ. In fig. S1, we visually depict the high-level view of this envisioned astrocytic computational support sub-circuit.

Full Algorithm Specification for Simulating a CSDP-Adapted SNN

In this section, we provide the pseudocode (Algorithm 1; see next page) required for simulating a recurrent SNN, of arbitrary depth, processing an input pattern over an arbitrary-length stimulus window. Note that Algorithm 1 depicts both supervised context-driven and unsupervised variations of our framework.

Algorithm 1 The contrastive-signal-dependent plasticity credit assignment algorithm. Lines in green font depict portions of the simulation code executed if supervised CSDP is to be utilized.

Input: sample $(\mathbf{y}, \mathbf{x})$ (with $\mathbf{y} = \emptyset$ if unsupervised), SNN parameters Θ, and
    virtual data label y_{type} (with value: $1 =$ "positive" and $0 =$ "negative")
Hyperparameters: SGD step size η, stimulus time T, integration constant Δt, time constants τ_m and τ_{tr},
    adaptive threshold step λ_v
// $\leftarrow$ denotes the overriding of a variable/object, $\Omega()$ is a bounding function
function SIMULATE($(\mathbf{y}, \mathbf{x}, y_{type}), \Theta$)
  for $t = 1$ to T **do**
    $\mathbf{s}^0(t) \sim \mathcal{B}(1, p = \mathbf{x}), \quad \mathbf{s}_y(t) = \mathbf{y}$ ▷ Sample sensory input and context to get an input spike at time t
    for $\ell = 1$ to L **do**
      // Compute the current and voltage components of layer ℓ
      if $\ell < L$ **then**
        $\mathbf{j}^\ell(t) = R_e\big(\mathbf{W}^\ell \cdot \mathbf{s}^{\ell-1}(t)\big) + R_e\big(\mathbf{V}^\ell \cdot \mathbf{s}^{\ell+1}(t)\big) - R_i\big((\mathbf{M}^\ell \odot (1 - \mathbf{I}^\ell)) \cdot \mathbf{s}^\ell(t)\big)$
      else
        $\mathbf{j}^\ell(t) = R_e\big(\mathbf{W}^\ell \cdot \mathbf{s}^{\ell-1}(t)\big) - R_i\big((\mathbf{M}^\ell \odot (1 - \mathbf{I}^\ell)) \cdot \mathbf{s}^\ell(t)\big)$
      if $\mathbf{y} \neq \emptyset$ **then**
        $\mathbf{j}^\ell(t) = \mathbf{j}^\ell(t) + R_e\big(\mathbf{B}^\ell \cdot \mathbf{s}_y(t)\big)$ ▷ Apply label context pressure (if supervised)
      $\mathbf{v}^\ell(t + \Delta t) = \mathbf{v}^\ell(t) + \frac{\Delta t}{\tau_m}\Big(- \mathbf{v}^\ell(t) + \mathbf{j}^\ell(t)\Big)$
      // Run the spike model given $\mathbf{j}(t)$ and $\mathbf{v}(t)$ (and depolarize $\mathbf{v}(t)$ if applicable)
      $\mathbf{s}^\ell(t) = \mathbf{v}^\ell(t + \Delta t) > v^\ell_{thr}, \quad \mathbf{v}^\ell(t) = \mathbf{v}^\ell(t + \Delta t) \odot (1 - \mathbf{s}^\ell(t)),$
      $v^\ell_{thr} = v^\ell_{thr} + \lambda_v\big((\sum_{j=1}^{J_\ell}, \; \mathbf{s}^\ell(t)_j) - 1\big)$
      // Update the activation trace for layer ℓ
      $\mathbf{z}^\ell_t = \mathbf{s}^\ell_t - \frac{\Delta t}{\tau_{tr}} \mathbf{z}^\ell_t \odot (1 - \mathbf{z}^\ell_t)$
      // Calculate local synaptic adjustments and update its synaptic efficacies
      Get loss $\mathcal{C}(\mathbf{z}^\ell(t), y_{type})$ & its partial derivative w.r.t. $\mathbf{z}^\ell(t)$, i.e., $\delta^\ell(t) = \frac{\partial \mathcal{L}_G(\mathbf{z}^\ell(t), y_{type})}{\partial \mathbf{z}^\ell(t)}$
      $\Delta \mathbf{W}^\ell = \Big(R_e \delta^\ell(t) \cdot \big(\mathbf{s}^{\ell-1}(t-1)\big)^T\Big) + \lambda_d\big(\mathbf{s}^\ell(t) \cdot (1 - \mathbf{s}^{\ell-1}(t))^T\big), \quad \mathbf{W}^\ell \leftarrow \Omega\big(\mathbf{W}^\ell - \eta \Delta \mathbf{W}^\ell\big)$
      $\Delta \mathbf{V}^\ell = \Big(R_e \delta^\ell(t) \cdot \big(\mathbf{s}^{\ell+1}(t-1)\big)^T\Big) + \lambda_d\big(\mathbf{s}^\ell(t) \cdot (1 - \mathbf{s}^{\ell+1}(t))^T\big), \quad \mathbf{V}^\ell \leftarrow \Omega\big(\mathbf{V}^\ell - \eta \Delta \mathbf{V}^\ell\big)$
      $\Delta \mathbf{M}^\ell = \Big(R_i \delta^\ell(t) \cdot \big(\mathbf{s}^{\ell}(t-1)\big)^T\Big) + \lambda_d\big(\mathbf{s}^\ell(t) \cdot (1 - \mathbf{s}^{\ell}(t))^T\big), \quad \mathbf{M}^\ell \leftarrow \Omega\big(\mathbf{M}^\ell - \eta \Delta \mathbf{M}^\ell\big)$
      if $\mathbf{y} \neq \emptyset$ **then**
        $\Delta \mathbf{B}^\ell = \Big(R_e \delta^\ell(t) \cdot \big(\mathbf{s}_y(t-1)\big)^T + \lambda_d\big(\mathbf{s}^\ell(t)\big) \cdot (1 - \mathbf{s}^y(t))^T\Big), \quad \mathbf{B}^\ell \leftarrow \Omega\big(\mathbf{B}^\ell - \eta \Delta \mathbf{B}^\ell\big)$
      // Run ℓth local predictor, error neurons and adjust its synaptic efficacies
      $\mathbf{v}^\ell_\mu(t + \Delta t) = \mathbf{v}^\ell_\mu(t) + \frac{\Delta t}{\tau_m}\Big(- \mathbf{v}^\ell_\mu(t) + R_e(\mathbf{G}^{\ell+1} \cdot \mathbf{s}^{\ell+1}(t))\Big)$
      $\mathbf{s}^\ell_\mu(t) = \mathbf{v}^\ell_\mu(t + \Delta t) > v^\ell_{thr,\mu},$
      $\mathbf{e}^\ell(t) = \mathbf{s}^\ell_\mu(t) - \mathbf{s}^\ell(t)$ ▷ Calculate the error/mismatch activities
      $\Delta \mathbf{G}^\ell = \big(R_e \mathbf{e}^{\ell-1}(t)\big) \cdot (\mathbf{s}^\ell(t))^T, \quad \mathbf{G}^\ell \leftarrow \Omega\big(\mathbf{G}^\ell - \eta \Delta \mathbf{G}^\ell\big)$
    if $\mathbf{y} \neq \emptyset$ **then**
      // Calculate classifier outputs and adjust its synaptic efficacies (if supervised)
      $\mathbf{v}_y(t + \Delta t) = \mathbf{v}_y(t) + \frac{\Delta t}{\tau_m}\big(- \mathbf{v}_y(t) + R_e(\sum_{\ell=1}^{L} \mathbf{A}^\ell \cdot \mathbf{s}^\ell(t))\big)$
      $\mu_y(t) = \mathbf{v}_y(t + \Delta t) > v^y_{thr}, \quad \mathbf{v}_y(t) = \mathbf{v}_y(t + \Delta t) \odot (1 - \mu_y(t))$
      $v^y_{thr} = \mu_y(t) + \lambda_v\big((\sum_{j=1}^{C} \mu_{y,j}(t) - 1\big)$
      $\Delta \mathbf{A}^\ell = R_e\big(\mu_y(t) - \mathbf{s}_y(t)\big) \cdot (\mathbf{s}^\ell(t))^T, \quad \mathbf{A}^\ell \leftarrow \Omega\big(\mathbf{A}^\ell - \eta \Delta \mathbf{A}^\ell\big), \; \forall \ell = 1 \ldots L$
    else
      $\{\mathbf{A}^\ell = \emptyset\}_{\ell=1}^{L}$ ▷ In unsupervised model, these parameters are set to empty/null values
  Return $\Theta = \{\mathbf{W}^\ell, \mathbf{V}^\ell, \mathbf{M}^\ell, \mathbf{B}^\ell\}_{\ell=1}^{L} \bigcup \{\mathbf{G}^\ell\}_{\ell=1}^{L} \bigcup \{\mathbf{A}^\ell\}_{\ell=1}^{L}$ ▷ Output newly updated model parameters

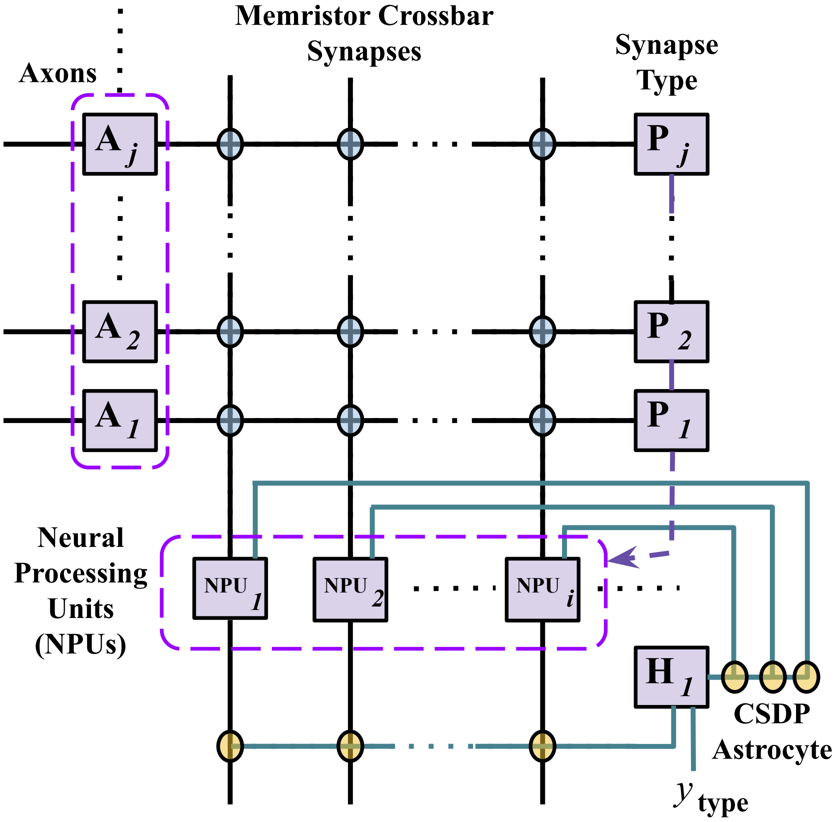

fig. S1. **A Neuromorphic Design Sketch of CSDP Astrocytic Computation.** A visualization of a neuromorphic unit implementing the synaptic transmission from layer $\ell - 1$ to layer ℓ. Hardware axons ($\mathbf{A}_j$) are represented as horizontal lines/rows, hardware dendrites are depicted as vertical lines/columns, and memristive synaptic connections are represented as row-column junctions (light transparent blue circles indicate plastic synaptic junctures while light transparent yellow circles indicate non-plastic synaptic junctures). Neuronal processing units (NPU_i), i.e.., hardware leaky integrate-and-fire spiking cells, receive input values from the dendrites while the hardware astrocyte $\mathbf{H}_1$ receives input from a non-plastic excitatory line from the outputs of the NPUs (in this case, their spike values but this could be modified to take in trace signals), as well as a single binary value indicating whether to operate in positive or negative modulation mode. This astrocyte recurrently emits a value to the NPUs to deposit the required contrastive signal (along an excitatory non-plastic line) to modulate the plastic STDP updates applied to synaptic strengths. Note we also depict, neuromorphically, additional units ($\mathbf{P}_j$) that transmit synaptic "types", which are discrete values representing whether a primary synapse is excitatory (e.g., a one) or inhibitory (e.g., a two) to the NPUs.

	MNIST		
**Layer Size**	**ACC** (%)	**BCE** (nats)	**NoPS**
$N_{\text{lif}} = (500, 100)$	93.65 ± 0.09	162.33 ± 0.59	$1,206,000$
$N_{\text{lif}} = (1000, 200)$	96.13 ± 0.11	147.15 ± 1.13	$3,232,000$
$N_{\text{lif}} = (2000, 400)$	97.21 ± 0.05	138.48 ± 0.41	$9,744,000$
$N_{\text{lif}} = (3000, 600)$	97.39 ± 0.05	134.68 ± 0.49	$19,536,000$
$N_{\text{lif}} = (4000, 800)$	97.46 ± 0.07	134.38 ± 0.12	$32,608,000$
$N_{\text{lif}} = (5000, 1000)$	97.58 ± 0.05	134.23 ± 0.59	$48,960,000$
$N_{\text{lif}} = (6000, 1200)$	97.63 ± 0.04	132.10 ± 0.31	$68,592,000$
$N_{\text{lif}} = (7000, 1400)$	97.67 ± 0.02	131.01 ± 0.19	$91,504,000$

table S1. **CSDP Model Performance with Respect to Number of Neurons.** Measurements, for different size of the hidden layers of the CSDP SNN architecture, of generalization accuracy (ACC, in terms of %; higher is better) and reconstruction binary cross entropy (BCE, in terms of nats; lower is better) on the MNIST database. Means and standard deviations for 5 trials are measured for each metric. The SNN model training setting was configured to be the same as the one for the model of Table 1 in the main paper (30 epochs, batch size of 500). Note: The number of plastic synapses (NoPS) reported here also includes both classification and reconstruction parameters since reconstruction efficacy were measured. The number of (total) synapses (NoS) would be equal to the NoPS in this case.

	MNIST	
**Model Variant**	**ACC** (%)	**BCE** (nats)
$\lambda_v =$ ✗, $\mathbf{V}^\ell =$ ✗, $\mathbf{B}^\ell =$ ✗	95.10 ± 0.42	159.05 ± 0.31
$\lambda_v =$ ✔, $\mathbf{V}^\ell =$ ✗, $\mathbf{B}^\ell =$ ✗	96.13 ± 0.24	140.11 ± 0.72
$\lambda_v =$ ✗, $\mathbf{V}^\ell =$ ✔, $\mathbf{B}^\ell =$ ✗	95.97 ± 0.32	146.02 ± 0.95
$\lambda_v =$ ✔, $\mathbf{V}^\ell =$ ✔, $\mathbf{B}^\ell =$ ✗	95.84 ± 0.03	147.91 ± 0.81
$\lambda_v =$ ✗, $\mathbf{V}^\ell =$ ✗, $\mathbf{B}^\ell =$ ✔	95.57 ± 0.57	141.12 ± 2.14
$\lambda_v =$ ✔, $\mathbf{V}^\ell =$ ✗, $\mathbf{B}^\ell =$ ✔	96.81 ± 0.05	138.05 ± 0.39
$\lambda_v =$ ✗, $\mathbf{V}^\ell =$ ✔, $\mathbf{B}^\ell =$ ✔	95.62 ± 0.11	153.51 ± 1.15
$\lambda_v =$ ✔, $\mathbf{V}^\ell =$ ✔, $\mathbf{B}^\ell =$ ✔	97.58 ± 0.05	134.23 ± 0.59

table S2. **Ablated CSDP Model Performance.** Measurements, for different ablations of the CSDP SNN architecture, of generalization accuracy (ACC, in terms of %; higher is better) and reconstruction binary cross entropy (BCE, in terms of nats; lower is better) on the MNIST dataset. The architecture and training process of each SNN model examined for this ablation experiment was configured to be the same as the one in Table 1 of the main paper, i.e., 5000 LIFs in first hidden layer and 1000 LIFs in the second hidden layer, with training carried out over 30 epochs with a batch size of 500 samples.

Item	Explanation
$\cdot$	Matrix/vector multiplication
$\odot$	Hadamard product (element-wise multiplication)
$\mathbf{z}_j$	jth scalar of vector $\mathbf{z}$
$\|\|\mathbf{v}\|\|_2$	Euclidean norm of vector $\mathbf{v}$
L	Number of layers
ℓ	A specific layer ℓ index ($0 \leq \ell \leq L$)
$\mathcal{C}()$	Local goodness contrastive function
$\delta(t)$	Goodness/contrastive modulatory signal
$\mathcal{E}()$	(Global) contrastive functional (goodness over time)
$\mathcal{F}()$	Optimization objective (contains goodness, reconstruction, classification)
$()^{\mathsf{T}}$	Transpose operation
$\mathbf{W}^{\ell}$	A matrix of synaptic weight values

table S3. **Definitions of mathematical operators and symbols.** Collected definitions of key mathematical symbols and operators used in this work.

Item	Explanation
ANN	Artificial neural network
DNN	Deep neural network
Backprop	Backpropagation of errors
NPU	Neural processing unit
BP-FNN	Backprop-trained feedforward network
SNN	Spiking neural network
STDP	Spike-timing-dependent plasticity
CSDP	Contrastive-signal-dependent plasticity
FF	Forward-forward
PFF	Predictive forward-forward
DRTP	Direct random target projection
BFA	Broadcast feedback alignment
L2-SigProp	L2-variant of signal propagation
Loc-Pred	Local predictors

table S4. **Abbreviation definitions.** Collected definitions of key abbreviations/acronyms used throughout this work.

Variable	Explanation	Plastic?
	Leaky Integrator Model Variables	
$\mathbf{j}^{\ell}$	Electrical current input to neurons in layer ℓ (vector w/ J_ℓ elements)	N/A (S,U)
$\mathbf{v}^{\ell}$	Membrane potential of neurons in layer ℓ (vector w/ J_ℓ elements)	N/A (S,U)
$\mathbf{s}^{\ell}$	Spikes emitted from neurons in layer ℓ (vector w/ J_ℓ elements)	N/A (S,U)
$\mathbf{z}^{\ell}$	Traces of neuronal activities in layer ℓ (vector w/ J_ℓ elements)	N/A (S,U)
$\mathbf{v}^{\ell}_{thr}$	Adaptive voltage thresholds for neurons in layer ℓ (vector w/ J_ℓ elements)	Yes (S,U)
$\delta(t)$	Modulator (vector) signal for neurons in layer ℓ (vector w/ J_ℓ elements)	N/A (S,U)
	Synaptic Model Variables	
$\mathbf{W}^{\ell}$	Bottom-up synaptic connections:<br>these propagate information from the layer $\ell - 1$ below	Yes (S,U)
$\mathbf{V}^{\ell}$	Top-down synaptic connections:<br>These propagate information from the layer $\ell + 1$ above	Yes (S,U)
$\mathbf{M}^{\ell}$	Lateral synaptic connections<br>These enforce lateral competition among neurons in ℓ (cross-layer inhibition)	Yes (S,U)
$\mathbf{I}^{\ell}$	Lateral identity masking matrix<br>$(1 - \mathbf{I}^{\ell})$ is used to enforce main diagonal of $\mathbf{M}^{\ell}$ to be zero (no self-excitation)	No (S,U)
$\mathbf{B}^{\ell}$	Context-mediating synaptic connections:<br>These propagate information from (optional) context/class signals	Yes (S)
$\mathbf{A}^{\ell}$	Classification synaptic connections:<br>These propagate information from a layer ℓ to a classification output layer	Yes (S,U)
$\mathbf{G}^{\ell}$	Generative synaptic connections:<br>These make a local prediction of layer $\ell - 1$'s activity given layer ℓ's current spikes	Yes (S,U)

table S5. **Specification of CSDP model variables.** Model variables, such as neuronal components or synaptic bundles are listed and explained here, with further indication as to whether each is plastic or not. “S“ denotes if a synaptic bundle variable is applicable only to the supervised variant of CSDP whereas “U“ indicates if it is applicable to the unsupervised variant.

Variable	Explanation	Value
Δt	Integration time constant	3 ms
T	Simulated time	$90 - 150$ ms
	Leaky Integrator Configuration	
τ_m	Membrane time constant	100 ms
R_E	Excitatory membrane resistance	0.1 (S), 0.1 (U) deciOhms
R_I	Inhibitory membrane resistance	0.035 (S), 0.01 (U) deciOhms
v^{ℓ}_{thr}	Voltage threshold	0.055 decivolts
λ_v	Threshold adaptation factor	0.001 decivolts
	CSDP Configuration	
θ_z	Goodness threshold	10 (S,U)
τ_{tr}	Variable trace time constant	13 ms
η_w	Global learning rate (Adam)	0.002
λ_d	Synaptic decay	0.00005
α	Negative sample mixing coefficient	0.5 (U)
$\mathbf{W}^{\ell}(0)$	All initial synapse conditions	$\sim \mathcal{U}(-1, 1)$
E	Number of epochs/passes through a dataset	30

table S6. **Specification of CSDP model constants**. Model constants that govern neuronal dynamics, plasticity in CSDP, or general simulation parameters are listed and explained here. “S“ indicates if a value chosen for a constant applies only to the supervised variant of CSDP while “U“ denotes if it applies only to the unsupervised variant. Unit of simulation time was milliseconds (ms).
